# A novel multivariate framework for functional gene networks enrichment analysis

**DOI:** 10.3389/fgene.2026.1871376

**Published:** 2026-07-24

**Authors:** Heewon Park, Seiya Imoto

**Affiliations:** 1 School of Mathematics, Statistics and Data Science, Sungshin Women’s University, Seoul, Republic of Korea; 2 Human Genome Center, Institute of Medical Science, University of Tokyo, Tokyo, Japan; 3 M&D Data Science Center, Institute of Science Tokyo, Tokyo, Japan

**Keywords:** cancer gene networks, enrichiment analysis, functional gene network analysis, immune pathway dysregulation, multivariate enrichment score

## Abstract

Gene network analysis is critically implicated in disease research for uncovering functional modules and interaction-driven pathways underlying biological and disease processes. However, the interpretation of large inferred networks remains challenging. Although functional gene network analysis allows the interpretation of large inferred networks, challenges such as reduction of multiple network-level features to a single composite score often limit their application. This data reduction can mask the important multivariate characteristics of gene networks, hindering efficient differentiation of individual contributions of distinct network components. Hence, this study aimed to investigate a novel computational strategy called Multivariate Framework for Functional Gene Network Enrichment Analysis (mFGNA). This framework incorporated diverse network-level features from a graph-theoretical perspective, including node properties (centrality), edge connectivity patterns (Jaccard distance), interaction strengths (edge weights), alongside traditional expression levels. Notably, mFGNA preserved these multidimensional characteristics, capturing complex rewiring of gene networks across different phenotypic states. Furthermore, mFGNA adopted a gene-level permutation strategy to evaluate the enrichment hypothesis, ensuring effective statistical inference and reduced computational complexity compared with phenotype-based permutations. Extensive Monte Carlo simulations validated mFGNA through both undirected and directed gene networks, showing consistently improved performance over existing approaches across diverse pathway settings. We also applied mFGNA to investigate immune pathway perturbations in cancer cell lines and identified significant network-level dysregulation in pancreatic and non-small cell lung cancers. Cancer-specific interaction modules were dominated by human leukocyte antigen class II genes. Meanwhile, normal cell networks were characterized by hub genes such as MMP1 and MMP3 that were implicated in tissue maintenance, highlighting immune remodeling in tumors and the potential molecular targets for developing diagnostic and therapeutic interventions. Overall, the study shows that mFGNA enables effective functional pathway discovery in complex gene networks, providing mechanistic insights and potential translational targets in disease contexts.

## 1 Introduction

Gene network analysis is crucial for investigating disease mechanisms, key drivers, and therapeutic targets, owing to its advantages in providing insights into functional gene modules and regulatory relationships often overlooked by single-gene analyses. Despite developing various computational methods for gene network inference, it is difficult to extract meaningful biological insights from large complex networks. Multiple studies on gene set enrichment analysis (GSEA) performed for the functional interpretation of gene sets, including overrepresentation analysis (ORA) (Draghici et al., 2003)and GSEA (Subramanian et al., 2005), have extended the research to gene network analyses. Functional network analysis links network structures with established pathways and functional processes to provide an effective framework for translating large networks into meaningful biological insights. Consequently, various enrichment approaches have been proposed to enhance this interpretation of the inferred networks. Signorelli et al. (2016) introduced the Network Enrichment Analysis Test (NEAT), which is an extension of the traditional over-representation analysis integrating gene–gene interactions and hypergeometric-based testing to assess connectivity-driven enrichment. In contrast, Gene Set Correlation Enrichment Analysis assesses the functional associations between differentially expressed and predefined genes by leveraging co-expression networks (Chang et al., 2024). Furthermore, Park et al. (2025) developed a gene behavior–based network enrichment approach that adapted GSEA to gene network settings by explicitly accounting for connectivity-driven gene behavior within the inferred networks.

**FIGURE 1:**
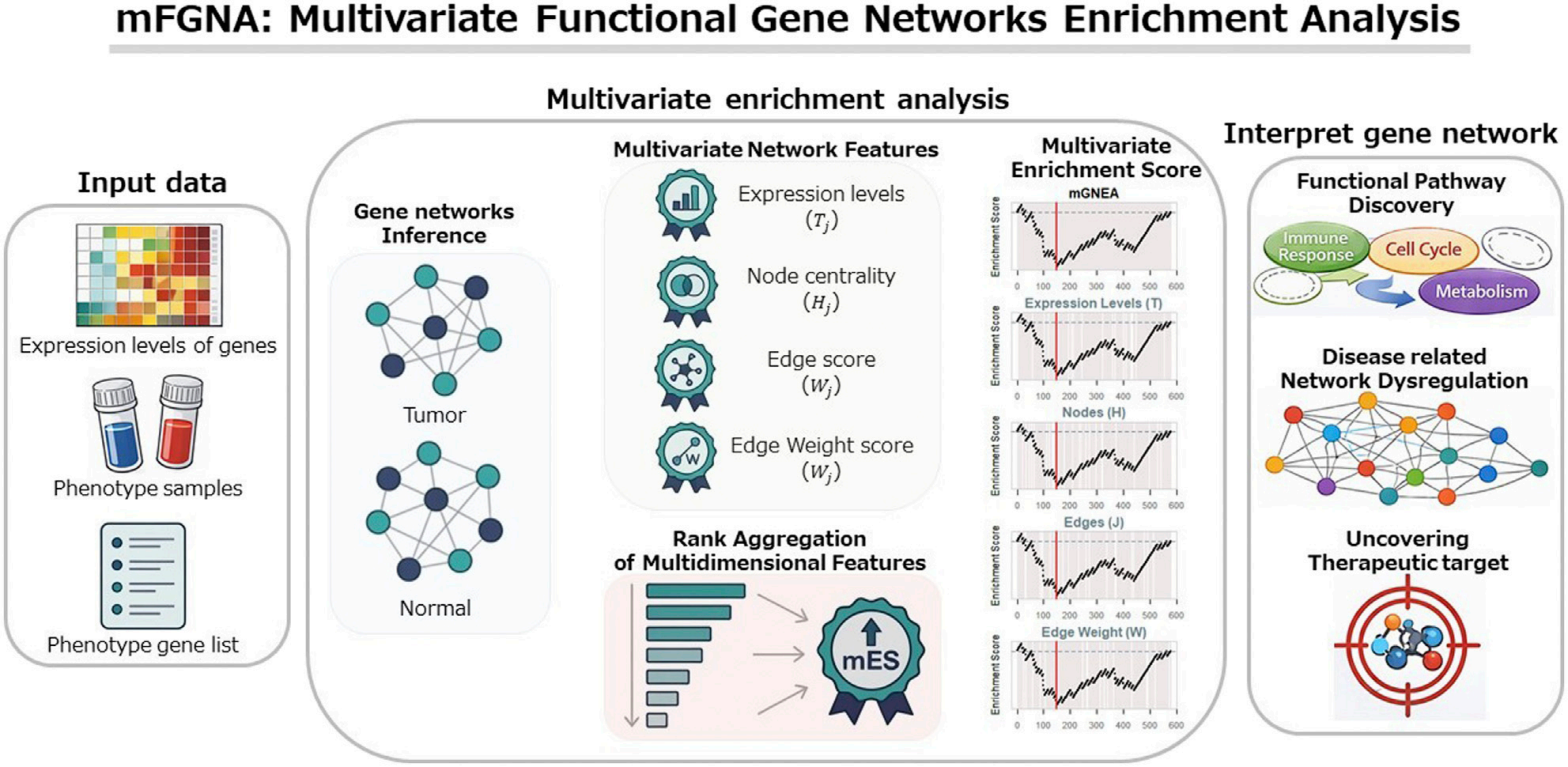
The overall framework of the proposed Multivariate Functional Gene Networks Enrichment Analysis (mFGNA).

However, such existing approaches present a major limitation of aggregating multiple network-level features into single composite scores before conducting a functional enrichment analysis. This can mask the important multivariate characteristics of gene networks, hindering effective differentiation of individual contributions of distinct network components. Tiong and Yeang (2019) reported that this inevitable loss of multivariate network information reduces biological interpretability of the overall functional gene analysis. Hence, to address these limitations, this study aimed to develop a novel computational strategy called Multivariate Framework for Functional Gene Network Enrichment Analysis (mFGNA). This framework incorporated various network-level features from a graph-theoretical perspective, including node properties (centrality), edge connectivity patterns (Jaccard distance), interaction strengths (edge weights), and traditional expression levels. These multi-dimensional characteristics were preserved by mFGNA, which captured the complex rewiring of gene networks across different phenotypic states, such as normal and cancerous cells. Moreover, mFGNA employs a gene-level permutation strategy to construct a null distribution that is directly aligned with the enrichment hypothesis, enabling more robust statistical inference. This approach resolves previously reported inconsistencies (Tian et al., 2005), where conventional GSEA and GbNEA generate null distributions through phenotype permutation, despite testing gene-set enrichment using a Kolmogorov–Smirnov statistic. Because phenotype permutation implicitly assumes no relevant genes in the query set, it creates a conceptual mismatch between the test statistic and the null model, which is more consistent with gene-set permutation. Notably, mFGNA permutes genes within a network, providing a null distribution consistent with the enrichment hypothesis and enabling a more coherent and reliable statistical inference for functional gene network enrichment analysis (GNEA). Furthermore, permuting genes, rather than phenotype labels, reduces computational complexity because it avoids repeated re-estimation of gene networks for each permutation. Figure 1 presents the overall framework of the proposed mFGNA.

The proposed mFGNA framework was evaluated through extensive Monte Carlo simulations and real-world cancer transcriptomic datasets. Simulation studies were conducted across diverse network structures and sizes to assess the ability of mFGNA to identify phenotype-associated molecular interplays. Furthermore, mFGNA was applied to investigate immune pathway perturbations across multiple cancer types and to identify network-level molecular signatures associated with cancer progression. These analyses demonstrate the utility of mFGNA as a general framework for interpreting complex biological networks and identifying disease-associated pathway perturbations.

In the Methods section, the proposed mFGNA framework has been introduced along with its evaluation through comprehensive Monte Carlo simulations.

## 2 A novel multivariate framework for functional gene networks enrichment analysis

### 2.1 Gene network estimation

We included two phenotypic groups, N and C , representing, for example, normal versus cancer cell lines or severe versus non-severe states. Suppose $X={\left(x_{1},\ldots ,x_{n}\right)}^{T}\in R^{n\times p}$ and $Y={\left(y_{1},\ldots ,y_{n}\right)}^{T}\in R^{n\times p}$ are n×p expression matrices of p genes in n samples for phenotypes N and C , respectively.

#### 2.1.1 Undirected gene network

A co-expression network was used to describe the undirected gene network; the correlation coefficient between the expression of the $j^{th}$ and $k^{th}$ genes was considered as the edge weight. The edge weight between genes was quantified using the Pearson correlation coefficient, as follows:

$$\gamma_{jk}=\frac{\sum_{i=1}^{n}\left(x_{ij}-{\bar{x}}_{j}\right)\left(x_{ik-{\bar{x}}_{k}}\right)}{\sum_{i=1}^{n}{\left(x_{ij}-{\bar{x}}_{j}\right)}^{2}\sum_{i=1}^{n}{\left(x_{ik}-{\bar{x}}_{k}\right)}^{2}},$$ (1)

where $x_{ij}$ and $x_{ik}$ denote the expression of the $j^{th}$ and $k^{th}$ genes in the $i^{th}$ sample, respectively; and ${\bar{x}}_{j}$ and ${\bar{x}}_{k}$ are the averages of the $j^{th}$ and $k^{th}$ genes, respectively.

#### 2.1.2 Directed gene network

We adopted the following linear regression framework to model directed gene-gene interactions:

$$x_{i\ell }=\overset{p}{\underset{j\neq \ell }{\sum }}x_{j}\beta_{\ell j}+\epsilon_{i\ell },\;\ell =1,\ldots ,p,$$ (2)

where $\beta_{\ell }={\left(\beta_{\ell 1},\ldots ,\beta_{\ell p}\right)}^{T}\in R^{p}$ denotes the edge-weight parameters linking the $\ell^{th}$ target gene to the remaining p−1 regulator genes, and $\epsilon_{\ell }={\left(\epsilon_{1\ell },\ldots ,\epsilon_{n\ell }\right)}^{T}\in R^{n}$ denotes a random error vector capturing unexplained variation. Typically, directed gene networks are inferred from high-dimensional gene expression data (i.e., several described in (Equation 2) genes and few samples). The following $L_{1}$ -type regularization method was Equation 3 adopted for network inference:

$${\hat{\beta }}_{\ell }=\arg\underset{\beta_{\ell }}{\min}\left\{\frac{1}{2}\overset{n}{\underset{i=1}{\sum }}{\left(x_{i\ell }-\overset{p}{\underset{j\neq \ell }{\sum }}x_{ij}\beta_{\ell j}\right)}^{2}+P_{\lambda }\left(\left|\beta_{\ell }\right|\right)\right\},$$ (3)

where $P_{\lambda }\left(\left|\beta_{\ell }\right|\right)$ denoted an $L_{1}$ -type penalty term (e.g., lasso (Tibshirani, 1996) and elastic net (Zou and Hastie, 2005), etc.).

### 2.2 Multiple topological features of the gene network

The estimated network was represented by a weighted graph G=(V,E,W) , where V={1,…,p} denoted vertices corresponding to p genes and E⊆V×V represented edges. An edge (i,j) indicated a molecular interaction between genes i and j , and $W=\left(w_{ij}\right),\;\left(i,j\right)\in E$ denoted the corresponding edge weights. Building on this graph, multiple topological features of the gene network were incorporated into the functional analysis by considering gene expression and network-derived characteristics, including node properties (i.e., V ), edge connectivity patterns (i.e., E ), and interaction strengths captured by edge weights (i.e., W ). We then proposed the mFGNA approach by extending the multivariate GSEA (Tiong and Yeang, 2019).

#### 2.2.1 Expression score

The t-statistic (Equation 4) was used to describe phenotype-specific transcriptomes such as normal and cancerous samples:

$$T_{j}=\frac{{\bar{x}}_{j}-{\bar{y}}_{j}}{S_{x_{j}y_{j}}\sqrt{\frac{1}{n_{N}}+\frac{1}{n_{C}}}},$$ (4)

where $S_{x_{j}y_{j}}=\sqrt{\left(n_{x_{j}}-1\right)s_{x_{j}}^{2}-\left(n_{y_{j}}-1\right)s_{y_{j}}^{2}}$ , $s_{x_{j}}$ $\left(s_{y_{j}}\right)$ and $n_{N}$ $\left(n_{C}\right)$ denote the standard deviation of $j^{th}$ gene expression and number of samples for the phenotype N and C , respectively.

#### 2.2.2 Node feature score

To characterize the centrality and influence of the nodes, we considered the following hubness measures for Equation 5 genes:

$$h_{j}^{N}=\frac{\left|V_{j}^{N}\right|}{\left|V_{j}^{N}\cup V_{j}^{C}\right|}\;and\;h_{j}^{C}=\frac{\left|V_{j}^{C}\right|}{\left|V_{j}^{N}\cup V_{j}^{C}\right|},$$ (5)

where $V_{j}^{N}$ and $V_{j}^{C}$ denoted node sets directly connected to the $j^{th}$ gene in the networks corresponding to the phenotypes N and C , respectively. The hubness measures $h_{j}^{N}$ and $h_{j}^{C}$ reflected the regulatory influence of the $j^{th}$ gene in the networks of phenotypes N and C , where higher hubness implied a stronger network-wide impact. We then define the score (Equation 6) of the $j^{th}$ node as follows:

$$H_{j}=h_{j}^{N}-h_{j}^{C}.$$ (6)

#### 2.2.3 Edge feature score

The Jaccard index $JI_{j}$ given in (Equation 7) (Li et al., 2014) was employed to quantify the similarity of edge structures between two networks of phenotypes, as follows:

$$JI_{j}=\frac{\left|E_{j}^{N}\cap E_{j}^{C}\right|}{\left|E_{j}^{N}\cup E_{j}^{C}\right|},$$ (7)

where $E_{j}^{N}$ and $E_{j}^{C}$ denoted the edge sets of the $j^{th}$ gene in the networks of the phenotypes C and N , respectively. Accordingly, we assessed edge-level differences using the corresponding Jaccard distance, Equation 8 defined as follows:

$$J_{j}=1-JI_{j}=1-\frac{\left|E_{j}^{N}\cap E_{j}^{C}\right|}{\left|E_{j}^{N}\cup E_{j}^{C}\right|}.$$ (8)

#### 2.2.4 Edge-weight score

We characterized the edge weights by quantifying the differences between the edge weight matrices $W^{C}$ and $W^{N}$ corresponding to phenotypes C and N , respectively.

- Undirected network:
$$W_{j}=\|W_{j}^{C}-W_{j}^{N}\|.$$ (9)
- Directed network:
  In directed network, the columns and rows of the edge weight matrix W corresponded to the target and regulator genes. Thus, the discrepancy between the two edge weight matrices was quantified as follows:
$$W_{j}=W_{j}^{\text{In}}+W_{j}^{\text{Out}},$$ (10)
  where
$$W_{j}^{\text{In}}=\|W_{\cdot j}^{C}-W_{\cdot j}^{N}\|\;and\;W_{j}^{\text{Out}}=\|W_{j\cdot }^{C}-W_{j\cdot }^{N}\|,$$
  where $W_{j\cdot }$ and $W_{\cdot j}$ denoted the $j^{th}$ row and column of W , respectively.

### 2.3 Enrichment score

Within the multi-dimensional framework, enrichment was measured based on the degree of over-representation of each network feature score (i.e., $T_{j},H_{j},J_{j}$ , and $W_{j}$ ) at the top or bottom of the ranked gene list. The enrichment score was then computed by integrating the multi-dimensional feature scores at those extremes.

- We ranked the genes in the node set V of the query gene network according to each score (i.e., *T_j_*, (Equation 4) *H_j_*, (Equation 6) *J_j_*, (Equation 8) and *W_j_* (Equations 9, 10)) separately to construct the corresponding ranked gene lists,
- Ranked gene list based on expression score:
$$L_{T}=\left\{g_{\left(1\right)}^{L_{T}},\ldots ,g_{\left(p\right)}^{L_{T}}\right\}$$
- Ranked gene list based on node feature score:
$$L_{H}=\left\{g_{\left(1\right)}^{L_{H}},\ldots ,g_{\left(p\right)}^{L_{H}}\right\}$$
- Ranked gene list based on edge feature score:
$$L_{J}=\left\{g_{\left(1\right)}^{L_{J}},\ldots ,g_{\left(p\right)}^{L_{J}}\right\}$$
- Ranked gene list based on edge weight score:
$$L_{W}=\left\{g_{\left(1\right)}^{L_{W}},\ldots ,g_{\left(p\right)}^{L_{W}}\right\}$$
- Let U(i) is the union gene set appearing at positions i or lower across the four ranked lists.
$$U\left(i\right)=\underset{L\in \left\{L_{T},L_{H},L_{J},L_{W}\right\}}{⋃}\left\{g_{\left(j\right)}^{L}|i\leq j\right\}$$
  where $g_{\left(j\right)}^{L}$ is the $j^{th}$ -ranked gene in the ranking list $L\in \left\{L_{T},L_{H},L_{J},L_{W}\right\}$ . We evaluated the gene fraction associated with a specific pathway by examining gene union belonging to the gene set $V^{*}$ involved in a specific pathway, i.e., $g\in \left\{U\left(i\right)\cap V^{*}\right\}$ , (Hit) and those outside, i.e., $g\in \left\{U\left(i\right)\backslash V^{*}\right\}$ (Miss) appearing up to position i in the ranked list $L\in \left\{L_{T},L_{H},L_{J},L_{W}\right\}$ ,
$$\begin{array}{c} Hit_{i}\left(V^{*}\right)=\underset{g\in \left\{U\left(i\right)\cap V^{*}\right\}}{\sum }\frac{1}{\left|V^{*}\right|},\\ Miss_{i}\left(V^{*}\right)=\underset{g\in \left\{U\left(i\right)\backslash V^{*}\right\}}{\sum }\frac{1}{\left|V|-|V^{*}\right|}. \end{array}$$
- The multi-dimensional enrichment score (mES) was defined as the maximum absolute deviation from zero of the following running-sum statistic across the ranked positions i ,
$$\Delta_{i}\left(V^{*}\right)=Hit_{i}\left(V^{*}\right)-Miss_{i}\left(V^{*}\right).$$
  Specifically, mES increases when the gene at the position i is contained in $V^{*}$ , and decreases when it is not.
- We further evaluated enrichment significance using a gene-level permutation framework rather than a phenotype permutation. This approach addresses the inconsistencies in conventional GSEA noted by Tian et al. (2005), where phenotype-based null distributions are misaligned with gene-based enrichment testing. We constructed a hypothesis-consistent null distribution by permuting genes within the network, which enabled a more coherent and reliable inference of gene network enrichment.

1. Genes in the node set V of the query gene network were randomly shuffled to generate a permuted pathway gene set $V_{\omega }^{*}$ .
2. We recompute $mES\left(V_{\omega }^{*}\right)$ for ω=1,…,Ω permutations. The observed enrichment score $mES\left(V^{*}\right)$ and the permuted scores $mES\left(V_{\omega }^{*}\right)$ were then normalized by separately rescaling positive and negative values. Specifically, each score was divided by the mean of the corresponding permutation-based null distribution of $mES\left(V_{\omega }^{*}\right)$ , yielding the normalized enrichment scores $mNES\left(V^{*}\right)$ and $mNES\left(V_{\omega }^{*}\right)$ .
3. Permutation p.value was computed as follows:
$$p.value=\frac{\sum_{\omega =1}^{\Omega }I\left(mNES\left(V_{\omega }^{*}\right)\geq mNES\left(V^{*}\right)\right)}{\Omega },$$
  where I(⋅) is an indicator function.
4. To account for multiple hypothesis testing, the Benjamini–Hochberg procedure was applied to control the false discovery rate (FDR) and provide the corresponding q-values (Benjamini and Hochberg, 1995).

Unlike conventional network enrichment approaches that perform enrichment analysis on a single ranked gene list derived from one network characteristic, the proposed mFGNA framework evaluates pathway enrichment using the union of genes identified across multiple ranked lists. Specifically, mFGNA constructs separate rankings based on expression differences, hubness differences, neighborhood similarity differences, and edge-weight differences, and subsequently integrates these rankings through the union set (U(i)) . Hit and Miss statistics are then calculated based on the cumulative contribution of genes appearing across the multiple rankings. This union-based enrichment framework preserves complementary network characteristics without reducing them to a single network-derived score prior to enrichment analysis. Consequently, mFGNA can identify pathway-level perturbations supported by multiple network dimensions and reduce the potential information loss associated with single-feature network representations.

The proposed permutation framework assumes that, under the null hypothesis, genes belonging to the query pathway are exchangeable with genes outside the pathway with respect to the multivariate network feature scores. Because each permuted gene set is constructed to have the same size as the query pathway, pathway-size effects are inherently controlled. Although gene-gene dependence is not explicitly modeled in the permutation procedure, such dependence is partially reflected through the network topology and interaction features used to derive the enrichment scores. The validity of the resulting permutation-based p-values was empirically evaluated through extensive true-negative simulation scenarios, where mFGNA consistently maintained high true-negative rates across diverse network settings.

### 2.4 Monte Carlo simulation 1 design

Gene expression data were obtained from the DepMap Public 22Q2 release. The dataset included 85 colorectal cancer, 48 gastric cancer, 156 non-small cell lung cancer, 77 small cell lung cancer, 83 breast cancer, and 103 non-cancerous cell lines. Non-cancerous cell lines were identified based on the “Non-Cancerous” annotation in the DepMap sample information file. Genes were ranked according to expression variance, and the highest-variance genes were selected to enrichment analysis. This strategy was adopted because variance-based gene filtering is commonly used in transcriptomic and gene network studies, where highly variable genes are selected prior to analysis (Wang et al., 2023; Cheng et al., 2020; Vengatharajuloo et al., 2023). Pathway genes were mapped using gene symbols, and only genes present in both the KEGG pathway database and the expression matrix were included in the analysis. For undirected networks, statistically significant pairwise correlations computed by using (Equation 1) (p.value <0.05 ) were retained as edges. For directed networks, the regularization parameters were selected using the Bayesian Information Criterion (BIC) (Zou et al., 2007).

For all cancer and normal cell lines, gene networks were constructed, resulting in a total of seven phenotype-specific gene networks. Each binary phenotype consisted of one cancer type versus a normal group. In colorectal cancer, the expression of 87 pathway-associated genes were considered in the enriched network, together with the remaining p−87 genes exhibiting the highest expression variance. Accordingly, colorectal cancer gene networks were inferred based on the expression profiles of these p genes. Next, multiple topological feature scores were calculated from the network differences between colorectal cancer and normal groups, followed by functional pathway analysis of the estimated gene network. Networks with p.values below the significance threshold α were considered as true-positive results of the GNEA. For the true-negative scenario, gene networks unrelated to the colorectal cancer pathway were estimated by selecting 5% of colorectal-associated genes (i.e., 4=87×0.05 genes) together with the remaining p−4 genes. In the true-negative scenario, two gene networks were estimated using the expression profiles of colorectal cancer and normal cell lines. If such a network was identified as significantly enriched in the colorectal cancer pathway, the result was regarded as a false positive in the enrichment analysis. A similar analysis was conducted for other cancer pathways (i.e., Gastric, SCL, NSCL, Breast, and Pancreatic cancers).

An additional true-negative scenario (mFGNA*) was constructed by preserving the original network structure and node composition while randomly permuting edge weights, hubness scores, Jaccard-distance scores, and expression-based scores across genes. The marginal distribution of each feature was maintained during randomization. Any significant enrichment identified from these randomized networks was regarded as a false positive. This scenario provides a more stringent evaluation of whether mFGNA captures genuine topological signals within gene networks.

## 3 Results

### 3.1 Monte Carlo Simulations 1

Monte Carlo simulations were conducted to validate the proposed strategy. The mFGNA was applied to the publicly available dataset of the Dependency Map (DepMap) portal (https://depmap.org/portal/), where expression data of various cancer cell lines were available.

The analysis focused on the pathways of Human Diseases, especially cancer-related pathways, in the Kyoto Encyclopedia of Genes and Genomes (KEGG) database (https://www.genome.jp/kegg/),

- Colerectal cancer (hsa05210; 87 genes)
- Gastric cancer (hsa05226; 150 genes)
- Small cell lung cancer (SCL) (hsa05222; 93 genes)
- Non small cell lung cancer (NSCL) (hsa05223; 73 genes)
- Breast cancer (hsa05224; 148 genes)
- Pancreatic cancer (hsa05212; 77 genes)

The performance of the proposed strategy was evaluated by comparing it with that of existing approaches, including GbNEA (Park et al., 2025), network-based GSEA (GSEAn), centrality-based GSEA (GSEAc) (Jung, 2024), ORA (Draghici et al., 2003), and NEAT (Signorelli et al., 2016). Because GbNEA is applicable only to the functional pathway analysis of undirected gene networks, mFGNA, GSEAn, GSEAc, ORA, and NEAT were used to evaluate directed networks. The existing gene set enrichment approaches were applied to the gene sets consisting of nodes within the inferred networks. We considered network sizes of p=500 , 1,000 , and 2,000 genes and a significance level of α=0.05 .

**FIGURE 2:**
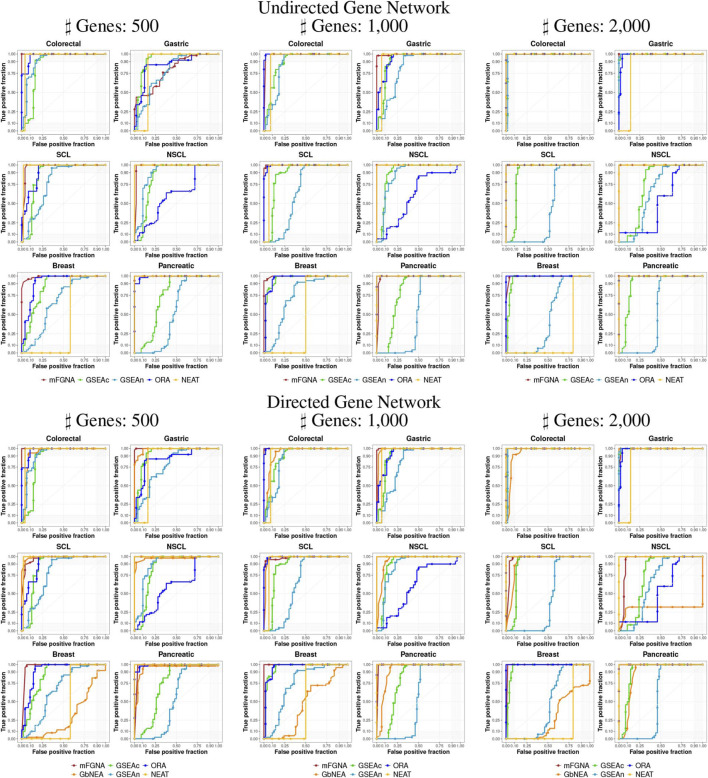
Receiver operating characteristic (ROC) curves comparing the performance of mFGNA, GSEAn, GSEAc, ORA, and NEAT for enriched gene network identification. Results are shown for networks constructed using 500, 1,000, and 2,000 genes across six cancer pathways. The upper panels represent undirected gene networks and the lower panels represent directed gene networks. Curves closer to the upper-left corner indicate superior discrimination performance.

Figure 2 presents the Receiver Operating Characteristic (ROC) curves for enriched gene network identification. Notably, all methods effectively identified enriched gene networks for the colorectal cancer pathway across different network sizes (Figure 2). However, the sensitivity of existing approaches was limited in the functional gene network analysis of breast cancer. Overall, although most existing methods performed reasonably well for certain cancer types, their accuracy was not consistent across pathways and network configurations. In contrast, mFGNA consistently achieved superior discrimination performance across all cancer-associated pathways, demonstrating its strong effectiveness against various cancer type and network size.

The proposed strategy was further evaluated using multiple performance metrics, including the area under the ROC curve (AUC), true positive rate (TPR), true negative rate (TNR), accuracy (ACC), precision (PREC), recall (REC), and the F1 measure (F1). Tables 1, 3 summarize the evaluation results for undirected and directed gene networks, respectively. As summarized in Tables 1, 2, mFGNA, GbNEA and ORA exhibited a strong overall performance regarding AUC across most cancer-related pathways. However, not only GSEAc and NEAT, but also GbNEA exhibited limited accuracy for breast cancer pathways. This was primarily attributed to low TPRs, indicating high tendency of failed detection of breast cancer pathway–enriched gene networks. Moreover, NEAT showed consistently weak performance in both undirected and directed network settings, largely because of its low TNRs, indicating a higher tendency to misclassify non-enriched networks as enriched. Notably, the consistently strong performance of mFGNA across all pathways, including the breast cancer pathways, may be attributed to its ability to integrate network-level topological differences, rather than relying solely on individual gene-level signals.

**TABLE 1:** Simulation results for functional enrichment analysis on undirected gene networks. mFGNA denotes the proposed method using the original network structure, whereas mFGNA* denotes the proposed method applied to perturbed networks. The symbol “–” denotes that precision is undefined due to the absence of selected pathways.

​	​	♯ genes: 500							♯ genes: 1000							♯ genes: 2000						
Cancer	Methods	AUC	ACC	TPR	TNR	PREC	REC	F1	AUC	ACC	TPR	TNR	PREC	REC	F1	AUC	ACC	TPR	TNR	PREC	REC	F1
Colorectal	mFGNA	1.00	1.00	1.00	1.00	1.00	1.00	1.00	0.99	0.99	1.00	0.98	0.98	1.00	0.99	1.00	1.00	1.00	1.00	1.00	1.00	1.00
	mFGNA*	1.00	0.95	1.00	0.90	0.91	1.00	0.95	0.99	0.93	1.00	0.86	0.88	1.00	0.93	0.97	0.91	1.00	0.82	0.85	1.00	0.92
	GSEAn	0.86	0.70	0.46	0.94	0.88	0.46	0.61	0.90	0.70	0.58	0.82	0.76	0.58	0.66	0.98	0.78	0.58	0.98	0.97	0.58	0.73
	GSEAc	0.91	0.71	0.58	0.84	0.78	0.58	0.67	0.86	0.82	0.82	0.82	0.82	0.82	0.82	0.98	0.97	1.00	0.94	0.94	1.00	0.97
	ORA	0.98	0.87	0.82	0.92	0.91	0.82	0.86	1.00	0.87	1.00	0.74	0.79	1.00	0.88	1.00	0.91	1.00	0.82	0.85	1.00	0.92
	NEAT	0.96	0.54	1.00	0.08	0.52	1.00	0.68	0.92	0.56	1.00	0.12	0.53	1.00	0.69	1.00	0.50	1.00	0.00	0.50	1.00	0.67
Gastric	mFGNA	0.79	0.64	0.28	1.00	1.00	0.28	0.44	1.00	0.98	0.96	1.00	1.00	0.96	0.98	1.00	1.00	1.00	1.00	1.00	1.00	1.00
	mFGNA*	0.78	0.59	0.28	0.90	0.74	0.28	0.41	0.94	0.93	0.96	0.90	0.91	0.96	0.93	1.00	0.96	1.00	0.92	0.93	1.00	0.96
	GSEAn	0.93	0.50	0.02	0.98	0.50	0.02	0.04	0.91	0.50	0.00	1.00	–	0.00	0.00	1.00	0.50	0.00	1.00	–	0.00	0.00
	GSEAc	0.77	0.67	0.38	0.96	0.90	0.38	0.54	0.81	0.75	0.60	0.90	0.86	0.60	0.71	1.00	0.89	0.78	1.00	1.00	0.78	0.88
	ORA	0.86	0.81	0.74	0.88	0.86	0.74	0.80	0.94	0.90	1.00	0.80	0.83	1.00	0.91	0.99	0.89	1.00	0.78	0.82	1.00	0.90
	NEAT	0.84	0.54	1.00	0.08	0.52	1.00	0.68	0.94	0.52	1.00	0.04	0.51	1.00	0.68	0.86	0.51	1.00	0.02	0.51	1.00	0.67
SCL	mFGNA	0.97	0.96	0.96	0.96	0.96	0.96	0.96	1.00	0.98	0.98	0.98	0.98	0.98	0.98	1.00	0.94	1.00	0.88	0.89	1.00	0.94
	mFGNA*	0.96	0.91	0.96	0.86	0.87	0.96	0.91	0.99	0.93	0.98	0.88	0.89	0.98	0.93	0.92	0.88	1.00	0.76	0.81	1.00	0.89
	GSEAn	0.86	0.50	0.02	0.98	0.50	0.02	0.04	0.87	0.48	0.00	0.96	0.00	0.00	0.00	0.88	0.45	0.00	0.90	0.00	0.00	0.00
	GSEAc	0.78	0.49	0.08	0.90	0.44	0.08	0.14	0.65	0.51	0.12	0.90	0.55	0.12	0.20	0.45	0.48	0.06	0.90	0.38	0.06	0.10
	ORA	0.91	0.76	0.70	0.82	0.80	0.70	0.74	0.99	0.91	1.00	0.82	0.85	1.00	0.92	1.00	0.96	1.00	0.92	0.93	1.00	0.96
	NEAT	0.98	0.53	1.00	0.06	0.52	1.00	0.68	0.94	0.50	1.00	0.00	0.50	1.00	0.67	1.00	0.50	1.00	0.00	0.50	1.00	0.67
NSCL	mFGNA	0.99	0.98	0.98	0.98	0.98	0.98	0.98	1.00	1.00	1.00	1.00	1.00	1.00	1.00	1.00	0.98	1.00	0.96	0.96	1.00	0.98
	mFGNA*	0.99	0.96	0.98	0.94	0.94	0.98	0.96	1.00	0.97	1.00	0.94	0.94	1.00	0.97	0.98	0.96	1.00	0.92	0.93	1.00	0.96
	GSEAn	0.84	0.84	0.88	0.80	0.81	0.88	0.85	0.90	0.84	0.98	0.70	0.77	0.98	0.86	0.74	0.68	1.00	0.36	0.61	1.00	0.76
	GSEAc	0.89	0.86	1.00	0.72	0.78	1.00	0.88	0.87	0.79	1.00	0.58	0.70	1.00	0.83	0.65	0.69	1.00	0.38	0.62	1.00	0.76
	ORA	0.60	0.60	0.64	0.56	0.59	0.64	0.62	0.60	0.68	0.86	0.50	0.63	0.86	0.73	0.52	0.58	1.00	0.16	0.54	1.00	0.70
	NEAT	1.00	0.74	1.00	0.48	0.66	1.00	0.79	1.00	0.72	1.00	0.44	0.64	1.00	0.78	1.00	0.55	1.00	0.10	0.53	1.00	0.69
Breast	mFGNA	0.99	0.95	0.92	0.98	0.98	0.92	0.95	1.00	0.97	0.94	1.00	1.00	0.94	0.97	0.99	0.98	1.00	0.96	0.96	1.00	0.98
	mFGNA*	0.93	0.90	0.92	0.88	0.88	0.92	0.90	0.96	0.90	0.94	0.86	0.87	0.94	0.90	0.99	0.96	1.00	0.92	0.93	1.00	0.96
	GSEAn	0.85	0.50	0.00	1.00	–	0.00	0.00	0.96	0.50	0.00	1.00	–	0.00	0.00	0.96	0.50	0.00	1.00	–	0.00	0.00
	GSEAc	0.66	0.51	0.04	0.98	0.67	0.04	0.08	0.74	0.52	0.06	0.98	0.75	0.06	0.11	0.46	0.49	0.00	0.98	0.00	0.00	0.00
	ORA	0.92	0.74	0.58	0.90	0.85	0.58	0.69	0.96	0.87	0.84	0.90	0.89	0.84	0.87	1.00	0.98	1.00	0.96	0.96	1.00	0.98
	NEAT	0.42	0.51	1.00	0.02	0.51	1.00	0.67	0.50	0.50	1.00	0.00	0.50	1.00	0.67	0.20	0.50	1.00	0.00	0.50	1.00	0.67
Pancreatic	mFGNA	1.00	1.00	1.00	1.00	1.00	1.00	1.00	0.98	0.97	1.00	0.94	0.94	1.00	0.97	1.00	1.00	1.00	1.00	1.00	1.00	1.00
	mFGNA*	0.99	0.93	1.00	0.86	0.88	1.00	0.93	0.95	0.89	1.00	0.78	0.82	1.00	0.90	0.97	0.94	1.00	0.88	0.89	1.00	0.94
	GSEAn	0.73	0.46	0.00	0.92	0.00	0.00	0.00	0.78	0.42	0.00	0.84	0.00	0.00	0.00	0.90	0.45	0.00	0.90	0.00	0.00	0.00
	GSEAc	0.53	0.48	0.04	0.92	0.33	0.04	0.07	0.51	0.45	0.04	0.86	0.22	0.04	0.07	0.54	0.48	0.00	0.96	0.00	0.00	0.00
	ORA	0.99	0.73	1.00	0.46	0.65	1.00	0.79	1.00	0.69	1.00	0.38	0.62	1.00	0.76	1.00	0.65	1.00	0.30	0.59	1.00	0.74
	NEAT	1.00	0.78	1.00	0.56	0.69	1.00	0.82	1.00	0.74	1.00	0.48	0.66	1.00	0.79	1.00	0.63	1.00	0.26	0.57	1.00	0.73
Average	mFGNA	0.96	0.92	0.86	0.99	0.99	0.86	0.89	0.99	0.98	0.98	0.98	0.98	0.98	0.98	1.00	0.98	1.00	0.97	0.97	1.00	0.98
	mFGNA*	0.94	0.87	0.86	0.89	0.87	0.86	0.84	0.97	0.93	0.98	0.87	0.88	0.98	0.93	0.97	0.94	1.00	0.87	0.89	1.00	0.94
	GSEAn	0.84	0.58	0.23	0.94	0.54	0.23	0.25	0.89	0.57	0.26	0.89	0.38	0.26	0.25	0.91	0.56	0.26	0.86	0.39	0.26	0.25
	GSEAc	0.76	0.62	0.35	0.89	0.65	0.35	0.39	0.74	0.64	0.44	0.84	0.65	0.44	0.45	0.68	0.67	0.47	0.86	0.49	0.47	0.45
	ORA	0.88	0.75	0.75	0.76	0.78	0.75	0.75	0.92	0.82	0.95	0.69	0.77	0.95	0.84	0.92	0.83	1.00	0.66	0.78	1.00	0.87
	NEAT	0.87	0.61	1.00	0.21	0.57	1.00	0.72	0.88	0.59	1.00	0.18	0.56	1.00	0.71	0.84	0.53	1.00	0.06	0.52	1.00	0.68

**TABLE 2:** Simulation results for functional enrichment analysis on undirected gene networks. mFGNA denotes the proposed method evaluated under the original simulation setting, whereas mFGNA* denotes the proposed method evaluated under a topology-preserving true negative scenario with randomized network features. The symbol “–” denotes that precision is undefined due to the absence of selected pathways and bold numbers indicate the best performance among the methods.

​	​	♯ genes: 500							♯ genes: 1000							♯ genes: 2000						
Cancer	Methods	AUC	ACC	TPR	TNR	PREC	REC	F1	AUC	ACC	TPR	TNR	PREC	REC	F1	AUC	ACC	TPR	TNR	PREC	REC	F1
Colorectal	mFGNA	**1.00**	**1.00**	1.00	1.00	1.00	1.00	1.00	0.99	**0.99**	1.00	0.98	0.98	1.00	0.99	**1.00**	**1.00**	1.00	1.00	1.00	1.00	1.00
	mFGNA*	1.00	0.95	1.00	0.90	0.91	1.00	0.95	0.99	0.93	1.00	0.86	0.88	1.00	0.93	0.97	0.91	1.00	0.82	0.85	1.00	0.92
	GSEAn	0.86	0.70	0.46	0.94	0.88	0.46	0.61	0.90	0.70	0.58	0.82	0.76	0.58	0.66	0.98	0.78	0.58	0.98	0.97	0.58	0.73
	GSEAc	0.91	0.71	0.58	0.84	0.78	0.58	0.67	0.86	0.82	0.82	0.82	0.82	0.82	0.82	0.98	0.97	1.00	0.94	0.94	1.00	0.97
	ORA	0.98	0.87	0.82	0.92	0.91	0.82	0.86	**1.00**	0.87	1.00	0.74	0.79	1.00	0.88	**1.00**	0.91	1.00	0.82	0.85	1.00	0.92
	NEAT	0.96	0.54	1.00	0.08	0.52	1.00	0.68	0.92	0.56	1.00	0.12	0.53	1.00	0.69	**1.00**	0.50	1.00	0.00	0.50	1.00	0.67
Gastric	mFGNA	0.79	0.64	0.28	1.00	1.00	0.28	0.44	**1.00**	**0.98**	0.96	1.00	1.00	0.96	0.98	**1.00**	**1.00**	1.00	1.00	1.00	1.00	1.00
	mFGNA*	0.78	0.59	0.28	0.90	0.74	0.28	0.41	0.94	0.93	0.96	0.90	0.91	0.96	0.93	1.00	0.96	1.00	0.92	0.93	1.00	0.96
	GSEAn	**0.93**	0.50	0.02	0.98	0.50	0.02	0.04	0.91	0.50	0.00	1.00	–	0.00	0.00	**1.00**	0.50	0.00	1.00	–	0.00	0.00
	GSEAc	0.77	**0.67**	0.38	0.96	0.90	0.38	0.54	0.81	0.75	0.60	0.90	0.86	0.60	0.71	**1.00**	0.89	0.78	1.00	1.00	0.78	0.88
	ORA	0.86	0.81	0.74	0.88	0.86	0.74	0.80	0.94	0.90	1.00	0.80	0.83	1.00	0.91	0.99	0.89	1.00	0.78	0.82	1.00	0.90
	NEAT	0.84	0.54	1.00	0.08	0.52	1.00	0.68	0.94	0.52	1.00	0.04	0.51	1.00	0.68	0.86	0.51	1.00	0.02	0.51	1.00	0.67
SCL	mFGNA	0.97	**0.96**	0.96	0.96	0.96	0.96	0.96	**1.00**	**0.98**	0.98	0.98	0.98	0.98	0.98	**1.00**	**0.94**	1.00	0.88	0.89	1.00	0.94
	mFGNA*	0.78	0.59	0.28	0.90	0.74	0.28	0.41	0.94	0.93	0.96	0.90	0.91	0.96	0.93	1.00	0.96	1.00	0.92	0.93	1.00	0.96
	GSEAn	0.86	0.50	0.02	0.98	0.50	0.02	0.04	0.87	0.48	0.00	0.96	0.00	0.00	0.00	0.88	0.45	0.00	0.90	0.00	0.00	0.00
	GSEAc	0.78	0.49	0.08	0.90	0.44	0.08	0.14	0.65	0.51	0.12	0.90	0.55	0.12	0.20	0.45	0.48	0.06	0.90	0.38	0.06	0.10
	ORA	0.91	0.76	0.70	0.82	0.80	0.70	0.74	0.99	0.91	1.00	0.82	0.85	1.00	0.92	**1.00**	0.96	1.00	0.92	0.93	1.00	0.96
	NEAT	**0.98**	0.53	1.00	0.06	0.52	1.00	0.68	0.94	0.50	1.00	0.00	0.50	1.00	0.67	**1.00**	0.50	1.00	0.00	0.50	1.00	0.67
NSCL	mFGNA	0.99	**0.98**	0.98	0.98	0.98	0.98	0.98	**1.00**	**1.00**	1.00	1.00	1.00	1.00	1.00	**1.00**	**0.98**	1.00	0.96	0.96	1.00	0.98
	mFGNA*	0.99	0.96	0.98	0.94	0.94	0.98	0.96	1.00	0.97	1.00	0.94	0.94	1.00	0.97	0.98	0.96	1.00	0.92	0.93	1.00	0.96
	GSEAn	0.84	0.84	0.88	0.80	0.81	0.88	0.85	0.90	0.84	0.98	0.70	0.77	0.98	0.86	0.74	0.68	1.00	0.36	0.61	1.00	0.76
	GSEAc	0.89	0.86	1.00	0.72	0.78	1.00	0.88	0.87	0.79	1.00	0.58	0.70	1.00	0.83	0.65	0.69	1.00	0.38	0.62	1.00	0.76
	ORA	0.60	0.60	0.64	0.56	0.59	0.64	0.62	0.60	0.68	0.86	0.50	0.63	0.86	0.73	0.52	0.58	1.00	0.16	0.54	1.00	0.70
	NEAT	**1.00**	0.74	1.00	0.48	0.66	1.00	0.79	**1.00**	0.72	1.00	0.44	0.64	1.00	0.78	**1.00**	0.55	1.00	0.10	0.53	1.00	0.69
Breast	mFGNA	**0.99**	**0.95**	0.92	0.98	0.98	0.92	0.95	**1.00**	**0.97**	0.94	1.00	1.00	0.94	0.97	0.99	**0.98**	1.00	0.96	0.96	1.00	0.98
	mFGNA*	0.93	0.90	0.92	0.88	0.88	0.92	0.90	0.96	0.90	0.94	0.86	0.87	0.94	0.90	0.99	0.96	1.00	0.92	0.93	1.00	0.96
	GSEAn	0.85	0.50	0.00	1.00	–	0.00	0.00	0.96	0.50	0.00	1.00	–	0.00	0.00	0.96	0.50	0.00	1.00	–	0.00	0.00
	GSEAc	0.66	0.51	0.04	0.98	0.67	0.04	0.08	0.74	0.52	0.06	0.98	0.75	0.06	0.11	0.46	0.49	0.00	0.98	0.00	0.00	0.00
	ORA	0.92	0.74	0.58	0.90	0.85	0.58	0.69	0.96	0.87	0.84	0.90	0.89	0.84	0.87	**1.00**	**0.98**	1.00	0.96	0.96	1.00	0.98
	NEAT	0.42	0.51	1.00	0.02	0.51	1.00	0.67	0.50	0.50	1.00	0.00	0.50	1.00	0.67	0.20	0.50	1.00	0.00	0.50	1.00	0.67
Pancreatic	mFGNA	**1.00**	**1.00**	1.00	1.00	1.00	1.00	1.00	0.98	**0.97**	1.00	0.94	0.94	1.00	0.97	**1.00**	**1.00**	1.00	1.00	1.00	1.00	1.00
	mFGNA*	0.99	0.93	1.00	0.86	0.88	1.00	0.93	0.95	0.89	1.00	0.78	0.82	1.00	0.90	0.97	0.94	1.00	0.88	0.89	1.00	0.94
	GSEAn	0.73	0.46	0.00	0.92	0.00	0.00	0.00	0.78	0.42	0.00	0.84	0.00	0.00	0.00	0.90	0.45	0.00	0.90	0.00	0.00	0.00
	GSEAc	0.53	0.48	0.04	0.92	0.33	0.04	0.07	0.51	0.45	0.04	0.86	0.22	0.04	0.07	0.54	0.48	0.00	0.96	0.00	0.00	0.00
	ORA	0.99	0.73	1.00	0.46	0.65	1.00	0.79	**1.00**	0.69	1.00	0.38	0.62	1.00	0.76	**1.00**	0.65	1.00	0.30	0.59	1.00	0.74
	NEAT	**1.00**	0.78	1.00	0.56	0.69	1.00	0.82	**1.00**	0.74	1.00	0.48	0.66	1.00	0.79	**1.00**	0.63	1.00	0.26	0.57	1.00	0.73
Average	mFGNA	**0.96**	**0.92**	0.86	0.99	0.99	0.86	0.89	**0.99**	**0.98**	0.98	0.98	0.98	0.98	0.98	**1.00**	**0.98**	1.00	0.97	0.97	1.00	0.98
	mFGNA*	0.94	0.87	0.86	0.89	0.87	0.86	0.84	0.97	0.93	0.98	0.87	0.88	0.98	0.93	0.97	0.94	1.00	0.87	0.89	1.00	0.94
	GSEAn	0.84	0.58	0.23	0.94	0.54	0.23	0.25	0.89	0.57	0.26	0.89	0.38	0.26	0.25	0.91	0.56	0.26	0.86	0.39	0.26	0.25
	GSEAc	0.76	0.62	0.35	0.89	0.65	0.35	0.39	0.74	0.64	0.44	0.84	0.65	0.44	0.45	0.68	0.67	0.47	0.86	0.49	0.47	0.45
	ORA	0.88	0.75	0.75	0.76	0.78	0.75	0.75	0.92	0.82	0.95	0.69	0.77	0.95	0.84	0.92	0.83	1.00	0.66	0.78	1.00	0.87
	NEAT	0.87	0.61	1.00	0.21	0.57	1.00	0.72	0.88	0.59	1.00	0.18	0.56	1.00	0.71	0.84	0.53	1.00	0.06	0.52	1.00	0.68

Furthermore, it can be seen though Tables 2, 3 that mFGNA* maintained strong performance across both undirected and directed gene networks and consistently outperformed most competing approaches. Although its performance was slightly lower than that of the original mFGNA, the results suggest that mFGNA captures meaningful topological signals rather than merely exploiting differences in pathway composition.

**TABLE 3:** Simulation results for functional enrichment analysis on directed gene networks. mFGNA denotes the proposed method evaluated under the original simulation setting, whereas mFGNA* denotes the proposed method evaluated under a topology-preserving true negative scenario with randomized network features. The symbol “–” denotes that precision is undefined due to the absence of selected pathways and bold numbers indicate the best performance among the methods.

​	​	♯ genes: 500							♯ genes: 1000							♯ genes: 2000						
Cancer	Methods	AUC	ACC	TPR	TNR	PREC	REC	F1	AUC	ACC	TPR	TNR	PREC	REC	F1	AUC	ACC	TPR	TNR	PREC	REC	F1
Colorectal	mFGNA	**1.00**	**1.00**	1.00	1.00	1.00	1.00	1.00	**1.00**	**0.99**	1.00	0.98	0.98	1.00	0.99	**1.00**	**0.97**	1.00	0.94	0.94	1.00	0.97
	mFGNA*	1.00	0.98	1.00	0.96	0.96	1.00	0.98	1.00	0.98	1.00	0.96	0.96	1.00	0.98	1.00	0.94	1.00	0.88	0.89	1.00	0.94
	GbNEA	0.94	0.84	0.76	0.92	0.90	0.76	0.83	0.94	0.75	0.56	0.94	0.90	0.56	0.69	0.94	0.88	0.82	0.94	0.93	0.82	0.87
	GSEAn	0.86	0.70	0.46	0.94	0.88	0.46	0.61	0.90	0.70	0.58	0.82	0.76	0.58	0.66	0.98	0.78	0.58	0.98	0.97	0.58	0.73
	GSEAc	0.91	0.71	0.58	0.84	0.78	0.58	0.67	0.86	0.82	0.82	0.82	0.82	0.82	0.82	0.98	**0.97**	1.00	0.94	0.94	1.00	0.97
	ORA	0.98	0.87	0.82	0.92	0.91	0.82	0.86	**1.00**	0.87	1.00	0.74	0.79	1.00	0.88	**1.00**	0.91	1.00	0.82	0.85	1.00	0.92
	NEAT	0.96	0.54	1.00	0.08	0.52	1.00	0.68	0.92	0.56	1.00	0.12	0.53	1.00	0.69	**1.00**	0.50	1.00	0.00	0.50	1.00	0.67
Gastric	mFGNA	**1.00**	**0.98**	1.00	0.96	0.96	1.00	0.98	**1.00**	**0.99**	1.00	0.98	0.98	1.00	0.99	**1.00**	**0.99**	1.00	0.98	0.98	1.00	0.99
	mFGNA*	0.99	0.96	1.00	0.92	0.93	1.00	0.96	1.00	0.96	1.00	0.92	0.93	1.00	0.96	0.97	0.92	1.00	0.84	0.86	1.00	0.93
	GbNEA	0.99	0.93	0.90	0.96	0.96	0.90	0.93	0.99	0.95	1.00	0.90	0.91	1.00	0.95	**1.00**	0.96	1.00	0.92	0.93	1.00	0.96
	GSEAn	0.93	0.50	0.02	0.98	0.50	0.02	0.04	0.91	0.50	0.00	1.00	–	0.00	0.00	**1.00**	0.50	0.00	1.00	–	0.00	0.00
	GSEAc	0.77	0.67	0.38	0.96	0.90	0.38	0.54	0.81	0.75	0.60	0.90	0.86	0.60	0.71	**1.00**	0.89	0.78	1.00	1.00	0.78	0.88
	ORA	0.86	0.81	0.74	0.88	0.86	0.74	0.80	0.94	0.90	1.00	0.80	0.83	1.00	0.91	0.99	0.89	1.00	0.78	0.82	1.00	0.90
	NEAT	0.84	0.54	1.00	0.08	0.52	1.00	0.68	0.94	0.52	1.00	0.04	0.51	1.00	0.68	0.86	0.51	1.00	0.02	0.51	1.00	0.67
SCL	mFGNA	0.98	**0.91**	0.92	0.90	0.90	0.92	0.91	**0.99**	0.94	0.96	0.92	0.92	0.96	0.94	0.97	0.94	1.00	0.88	0.89	1.00	0.94
	mFGNA*	0.98	0.93	0.92	0.94	0.94	0.92	0.93	0.99	0.93	0.96	0.90	0.91	0.96	0.93	0.99	0.94	1.00	0.88	0.89	1.00	0.94
	GbNEA	**0.99**	0.94	0.98	0.90	0.91	0.98	0.94	0.98	**0.97**	0.98	0.96	0.96	0.98	0.97	0.93	0.93	1.00	0.86	0.88	1.00	0.93
	GSEAn	0.86	0.50	0.02	0.98	0.50	0.02	0.04	0.87	0.48	0.00	0.96	0.00	0.00	0.00	0.88	0.45	0.00	0.90	0.00	0.00	0.00
	GSEAc	0.78	0.49	0.08	0.90	0.44	0.08	0.14	0.65	0.51	0.12	0.90	0.55	0.12	0.20	0.45	0.48	0.06	0.90	0.38	0.06	0.10
	ORA	0.91	0.76	0.70	0.82	0.80	0.70	0.74	0.99	0.91	1.00	0.82	0.85	1.00	0.92	**1.00**	**0.96**	1.00	0.92	0.93	1.00	0.96
	NEAT	0.98	0.53	1.00	0.06	0.52	1.00	0.68	0.94	0.50	1.00	0.00	0.50	1.00	0.67	**1.00**	0.50	1.00	0.00	0.50	1.00	0.67
NSCL	mFGNA	**1.00**	**0.99**	1.00	0.98	0.98	1.00	0.99	**1.00**	**0.99**	1.00	0.98	0.98	1.00	0.99	0.94	0.94	0.96	0.92	0.92	0.96	0.94
	mFGNA*	1.00	0.95	1.00	0.90	0.91	1.00	0.95	1.00	0.94	1.00	0.88	0.89	1.00	0.94	0.99	0.96	0.96	0.96	0.96	0.96	0.96
	GbNEA	0.98	0.91	0.98	0.84	0.86	0.98	0.92	0.95	0.92	0.94	0.90	0.90	0.94	0.92	0.30	0.55	0.32	0.78	0.59	0.32	0.42
	GSEAn	0.84	0.84	0.88	0.80	0.81	0.88	0.85	0.90	0.84	0.98	0.70	0.77	0.98	0.86	0.74	0.68	1.00	0.36	0.61	1.00	0.76
​	GSEAc	0.89	0.86	1.00	0.72	0.78	1.00	0.88	0.87	0.79	1.00	0.58	0.70	1.00	0.83	0.65	0.69	1.00	0.38	0.62	1.00	0.76
	ORA	0.60	0.60	0.64	0.56	0.59	0.64	0.62	0.60	0.68	0.86	0.50	0.63	0.86	0.73	0.52	0.58	1.00	0.16	0.54	1.00	0.70
	NEAT	**1.00**	0.74	1.00	0.48	0.66	1.00	0.79	**1.00**	0.72	1.00	0.44	0.64	1.00	0.78	**1.00**	0.55	1.00	0.10	0.53	1.00	0.69
Breast	mFGNA	0.98	**0.97**	1.00	0.94	0.94	1.00	0.97	**1.00**	**0.98**	1.00	0.96	0.96	1.00	0.98	0.99	0.96	1.00	0.92	0.93	1.00	0.96
	mFGNA*	1.00	0.96	1.00	0.92	0.93	1.00	0.96	1.00	0.95	1.00	0.90	0.91	1.00	0.95	0.99	0.92	1.00	0.84	0.86	1.00	0.93
	GbNEA	0.30	0.48	0.00	0.96	0.00	0.00	0.00	0.45	0.45	0.00	0.90	0.00	0.00	0.00	0.27	0.43	0.00	0.86	0.00	0.00	0.00
	GSEAn	0.85	0.50	0.00	1.00	–	0.00	0.00	0.96	0.50	0.00	1.00	–	0.00	0.00	0.96	0.50	0.00	1.00	–	0.00	0.00
	GSEAc	0.66	0.51	0.04	0.98	0.67	0.04	0.08	0.74	0.52	0.06	0.98	0.75	0.06	0.11	0.46	0.49	0.00	0.98	0.00	0.00	0.00
	ORA	0.92	0.74	0.58	0.90	0.85	0.58	0.69	0.96	0.87	0.84	0.90	0.89	0.84	0.87	**1.00**	**0.98**	1.00	0.96	0.96	1.00	0.98
	NEAT	0.42	0.51	1.00	0.02	0.51	1.00	0.67	0.50	0.50	1.00	0.00	0.50	1.00	0.67	0.20	0.50	1.00	0.00	0.50	1.00	0.67
Pancreatic	mFGNA	0.98	0.93	1.00	0.86	0.88	1.00	0.93	0.99	**0.98**	1.00	0.96	0.96	1.00	0.98	**1.00**	**0.99**	1.00	0.98	0.98	1.00	0.99
	mFGNA*	0.98	0.95	1.00	0.90	0.91	1.00	0.95	0.99	0.96	1.00	0.92	0.93	1.00	0.96	1.00	0.96	1.00	0.92	0.93	1.00	0.96
	GbNEA	0.95	0.89	0.88	0.90	0.90	0.88	0.89	0.91	0.73	0.54	0.92	0.87	0.54	0.67	0.87	0.52	0.14	0.90	0.58	0.14	0.23
	GSEAn	0.73	0.46	0.00	0.92	0.00	0.00	0.00	0.78	0.42	0.00	0.84	0.00	0.00	0.00	0.90	0.45	0.00	0.90	0.00	0.00	0.00
	GSEAc	0.53	0.48	0.04	0.92	0.33	0.04	0.07	0.51	0.45	0.04	0.86	0.22	0.04	0.07	0.54	0.48	0.00	0.96	0.00	0.00	0.00
	ORA	0.99	0.73	1.00	0.46	0.65	1.00	0.79	**1.00**	0.69	1.00	0.38	0.62	1.00	0.76	**1.00**	0.65	1.00	0.30	0.59	1.00	0.74
	NEAT	**1.00**	0.78	1.00	0.56	0.69	1.00	0.82	**1.00**	0.74	1.00	0.48	0.66	1.00	0.79	**1.00**	0.63	1.00	0.26	0.57	1.00	0.73
Average	mFGNA	**0.99**	**0.96**	0.99	0.94	0.94	0.99	0.96	**1.00**	**0.98**	0.99	0.96	0.96	0.99	0.98	0.98	**0.97**	0.99	0.94	0.94	0.99	0.97
	mFGNA*	0.99	0.96	0.99	0.92	0.93	0.99	0.96	1.00	0.95	0.99	0.91	0.92	0.99	0.96	0.99	0.94	0.99	0.89	0.90	0.99	0.94
	GbNEA	0.86	0.83	0.75	0.91	0.75	0.75	0.75	0.87	0.80	0.67	0.92	0.76	0.67	0.70	0.72	0.71	0.55	0.88	0.65	0.55	0.57
	GSEAn	0.84	0.58	0.23	0.94	0.54	0.23	0.25	0.89	0.57	0.26	0.89	0.38	0.26	0.25	0.91	0.56	0.26	0.86	0.39	0.26	0.25
	GSEAc	0.76	0.62	0.35	0.89	0.65	0.35	0.39	0.74	0.64	0.44	0.84	0.65	0.44	0.45	0.68	0.67	0.47	0.86	0.49	0.47	0.45
	ORA	0.88	0.75	0.75	0.76	0.78	0.75	0.75	0.92	0.82	0.95	0.69	0.77	0.95	0.84	0.92	0.83	1.00	0.66	0.78	1.00	0.87
	NEAT	0.87	0.61	1.00	0.21	0.57	1.00	0.72	0.88	0.59	1.00	0.18	0.56	1.00	0.71	0.84	0.53	1.00	0.06	0.52	1.00	0.68

**FIGURE 3:**
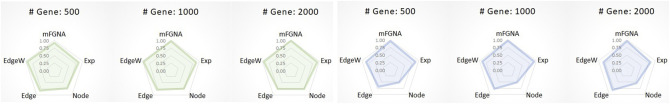
Ablation analysis of mFGNA across undirected and directed gene networks containing 500, 1,000, and 2,000 genes. Radar plots summarize the accuracy obtained using expression score alone (Exp), node feature score alone (Node), edge feature score alone (Edge), edge-weight score alone (EdgeW), and the full mFGNA framework.

Finally, we performed an ablation analysis to evaluate the contribution of each component of mFGNA, including expression score (Exp), node feature score (Node), edge feature score (Edge), edge-weight score (EdgeW). Figure 3 summarizes the accuracy obtained using each individual component and the full mFGNA framework across networks containing 500, 1,000, and 2,000 genes. In both undirected and directed network settings, the full mFGNA consistently achieved the highest accuracy regardless of network size. Among the individual components, expression information generally exhibited the strongest predictive performance, whereas node-, edge-, and edge-weight–based features provided complementary but comparatively weaker signals. Notably, integrating all four components resulted in a substantial improvement over any single component alone, demonstrating that each network characteristic contributes unique information for identifying phenotype-associated molecular interplays. These results support the design of mFGNA and indicate that its superior performance arises from the joint utilization of expression patterns, network topology, connectivity structure, and interaction strengths.

Overall, these results highlight the efficacy of mFGNA, which maintains a consistently high discriminative power across diverse pathway contexts and network structures.

In our analysis, gene network enrichment analysis was performed using genes exhibiting high expression variance. To assess the impact of variance-based gene selection, we additionally conducted enrichment analyses using 500 genes without applying variance-based filtering. The corresponding results are presented in Supplementary Table S1. As shown in the supplementary results, the performance of all evaluated methods decreased substantially under this setting. These findings indicate that the use of highly variable genes improves the stability and discriminative ability of network-based enrichment analyses and that the observed performance gains were not specific to mFGNA. Therefore, we recommend variance-based gene filtering as a preprocessing step for network-based enrichment analyses.

### 3.2 Monte Carlo Simulations 2

To further evaluate the robustness and generalizability of mFGNA on an independent cohort, additional Monte Carlo simulations were conducted using primary tumor samples from The Cancer Genome Atlas (TCGA). Unlike the DepMap-based simulations, which were performed using cancer cell-line expression profiles, the TCGA analysis was based on patient-derived tumor and matched normal tissue samples, thereby providing an independent validation dataset. TCGA cohorts corresponding to four cancer types that were also available in the DepMap analysis were considered: colorectal cancer (TCGA-COAD), gastric cancer (TCGA-STAD), breast cancer (TCGA-BRCA), and non-small-cell lung cancer (NSCLC), where NSCLC samples were constructed by combining lung adenocarcinoma (TCGA-LUAD) and lung squamous cell carcinoma (TCGA-LUSC) cohorts. Gene expression profiles were obtained from The Cancer Genome Atlas (TCGA) through the TCGAbiolinks package in R. For each cancer type, tumor and matched normal tissue samples were extracted to construct phenotype-specific expression datasets.

Similar to Monte Carlo Simulation 1, the methods were evaluated under both true-positive and true-negative scenarios. True-positive networks were constructed using genes belonging to the corresponding KEGG cancer pathway, whereas true-negative networks were generated using only a small proportion of pathway genes together with non-pathway genes. The inferred gene networks were subsequently subjected to functional enrichment analysis, and the performance of each method was evaluated using AUC, TPR, TNR, ACC, precision, recall, and F1-score. This design enabled a direct assessment of the reproducibility and generalizability of mFGNA in an independent patient-derived tumor dataset. Table 4 shows the results based on TCGA dataset.

**TABLE 4:** Independent validation of functional enrichment analysis methods using TCGA primary tumor and matched normal tissue samples. Gene networks were estimated from colorectal, gastric, non-small-cell lung (NSCL), and breast cancer cohorts, and enrichment performance was evaluated under true-positive and true-negative scenarios. The symbol “N/A” indicates that the corresponding result is unavailable because GbNEA is applicable only to undirected gene networks and cannot be applied to directed gene networks. The bold numbers indicate the best performance among the methods.

​	​	Undirected gene network							Directed gene network						
Cancer	Methods	AUC	ACC	TPR	TNR	Prec	Rec	F1	AUC	ACC	TPR	TNR	Prec	Rec	F1
Colorectal	mFGNA	**1.00**	**1.00**	1.00	1.00	1.00	1.00	1.00	**1.00**	**0.97**	1.00	0.94	0.94	1.00	0.97
	GbNEA	0.87	0.66	0.38	0.94	0.86	0.38	0.53	N/A	N/A	N/A	N/A	N/A	N/A	N/A
	GSEAn	0.92	0.90	1.00	0.80	0.83	1.00	0.91	0.92	0.90	1.00	0.80	0.83	1.00	0.91
	GSEAc	0.92	0.85	1.00	0.70	0.77	1.00	0.87	0.92	0.85	1.00	0.70	0.77	1.00	0.87
	ORA	**1.00**	0.94	1.00	0.88	0.89	1.00	0.94	**1.00**	0.94	1.00	0.88	0.89	1.00	0.94
	NEAT	**1.00**	0.89	1.00	0.78	0.82	1.00	0.90	**1.00**	0.89	1.00	0.78	0.82	1.00	0.90
Gastric	mFGNA	**1.00**	**0.99**	1.00	0.98	0.98	1.00	0.99	**1.00**	**0.95**	1.00	0.90	0.91	1.00	0.95
	GbNEA	0.94	0.89	0.94	0.84	0.85	0.94	0.90	N/A	N/A	N/A	N/A	N/A	N/A	N/A
	GSEAn	**1.00**	0.92	1.00	0.84	0.86	1.00	0.93	1.00	0.92	1.00	0.84	0.86	1.00	0.93
	GSEAc	0.99	0.85	1.00	0.70	0.77	1.00	0.87	0.99	0.85	1.00	0.70	0.77	1.00	0.87
	ORA	1.00	0.93	1.00	0.86	0.88	1.00	0.93	1.00	0.93	1.00	0.86	0.88	1.00	0.93
	NEAT	**1.00**	0.95	1.00	0.90	0.91	1.00	0.95	**1.00**	**0.95**	1.00	0.90	0.91	1.00	0.95
NSCL	mFGNA	**1.00**	**1.00**	1.00	1.00	1.00	1.00	1.00	**1.00**	**0.97**	1.00	0.94	0.94	1.00	0.97
	GbNEA	0.94	0.86	0.86	0.86	0.86	0.86	0.86	N/A	N/A	N/A	N/A	N/A	N/A	N/A
	GSEAn	0.72	0.66	0.60	0.72	0.68	0.60	0.64	0.72	0.66	0.60	0.72	0.68	0.60	0.64
	GSEAc	0.72	0.84	1.00	0.68	0.76	1.00	0.86	0.72	0.84	1.00	0.68	0.76	1.00	0.86
	ORA	0.97	0.77	1.00	0.54	0.68	1.00	0.81	0.97	0.77	1.00	0.54	0.68	1.00	0.81
	NEAT	**1.00**	0.96	1.00	0.92	0.93	1.00	0.96	**1.00**	0.96	1.00	0.92	0.93	1.00	0.96
Breast	mFGNA	**0.97**	0.84	1.00	0.68	0.76	1.00	0.86	0.99	**0.98**	1.00	0.96	0.96	1.00	0.98
	GbNEA	0.89	0.66	0.38	0.94	0.86	0.38	0.53	N/A	N/A	N/A	N/A	N/A	N/A	N/A
	GSEAn	0.95	**0.92**	1.00	0.84	0.86	1.00	0.93	0.95	0.92	1.00	0.84	0.86	1.00	0.93
	GSEAc	0.96	0.91	1.00	0.82	0.85	1.00	0.92	0.96	0.91	1.00	0.82	0.85	1.00	0.92
	ORA	**1.00**	0.79	1.00	0.58	0.70	1.00	0.83	**1.00**	0.79	1.00	0.58	0.70	1.00	0.83
	NEAT	**1.00**	0.97	1.00	0.94	0.94	1.00	0.97	**1.00**	0.97	1.00	0.94	0.94	1.00	0.97
Average	mFGNA	0.99	**0.96**	1.00	0.92	0.93	1.00	0.96	**1.00**	**0.97**	1.00	0.94	0.94	1.00	0.97
	GbNEA	0.91	0.77	0.64	0.90	0.86	0.64	0.70	N/A	N/A	N/A	N/A	N/A	N/A	N/A
	GSEAn	0.90	0.85	0.90	0.80	0.81	0.90	0.85	0.90	0.85	0.90	0.80	0.81	0.90	0.85
	GSEAc	0.90	0.86	1.00	0.73	0.79	1.00	0.88	0.90	0.86	1.00	0.73	0.79	1.00	0.88
	ORA	0.99	0.86	1.00	0.72	0.79	1.00	0.88	0.99	0.86	1.00	0.72	0.79	1.00	0.88
	NEAT	**1.00**	0.94	1.00	0.89	0.90	1.00	0.95	**1.00**	0.94	1.00	0.89	0.90	1.00	0.95

As summarized in Table 4, mFGNA exhibited consistently effective performance across most cancer types and network settings. Although the best performance varied depending on the evaluation metric and competing method, mFGNA generally achieved the highest or near-highest values for AUC and ACC while maintaining perfect sensitivity and high specificity. For undirected gene networks, mFGNA yielded the highest average ACC (0.96) while maintaining an average AUC of 0.99. In particular, mFGNA achieved perfect AUC and ACC values for colorectal and NSCL cancers and showed near-perfect performance for gastric cancer. Although NEAT achieved a slightly higher average AUC (1.00), its average ACC (0.94) remained lower than that of mFGNA. Similar trends were observed for directed gene networks, where mFGNA achieved the highest average ACC (0.97) together with the highest average AUC (1.00). Across individual cancer types, mFGNA consistently exhibited perfect sensitivity, while maintaining high specificity (TNR = 0.92 and 0.94 on average for undirected and directed networks, respectively). In contrast, GbNEA showed substantially reduced sensitivity despite relatively high specificity, whereas GSEAn, GSEAc, and ORA exhibited lower overall accuracies, particularly for NSCL and breast cancer pathways. Furthermore, GbNEA could not be applied to directed gene networks because the method is specifically designed for undirected network structures.

Overall, the TCGA-based validation results were highly consistent with those obtained from the DepMap analyses, demonstrating that the superior performance of mFGNA is preserved in an independent cohort of primary tumor samples. These findings support the robustness and generalizability of the proposed framework across different transcriptomic datasets and network construction settings.

### 3.3 Network-driven immune pathway dysregulation in cancer cells

Immune disease–associated pathways are critically implicated in cancer initiation and progression because dysregulated immune responses can promote chronic inflammation, impair immune surveillance, and facilitate tumor immune evasion (Tufail et al., 2025). Particularly, altered immune regulatory mechanisms are increasingly being recognized as key drivers of cancer development and therapeutic resistance (Liu et al., 2024).

To systematically characterize immune dysregulation associated with cancer, mFGNA was applied to investigate immune pathway perturbations in cancer cells. Immune disease-associated KEGG pathways were examined and grouped into eight categories, as follows: Asthma, Systemic lupus erythematosus, Rheumatoid arthritis, Autoimmune thyroid disease, Inflammatory bowel disease, Allograft rejection, Graft-versus-host disease, and Primary immunodeficiency. Table 5 summarizes the immune disease pathways, including the entries and numbers of genes involved in each pathway.

**TABLE 5:** Immune disease–associated KEGG pathways included in the enrichment analysis.

Name	Entry	# Genes
Asthma	05310	32
Systemic lupus erythematosus	05322	141
Rheumatoid arthritis	05323	95
Autoimmune thyroid disease	05320	54
Inflammatory bowel disease	05321	66
Allograft rejection	05330	39
Graft-versus-host disease	05332	45
Primary immunodeficiency	05340	38

A total of 332 genes involved in immune disease-related pathways were used to construct gene networks for cancer and normal cell lines. They were inferred from gene expression profiles of the DepMap dataset used in Monte Carlo simulations. For each cancer versus normal cell comparison, two immune gene networks corresponding to binary phenotypes were estimated. Next, we applied the proposed mFGNA to identify the dysregulated immune disease pathways in cancer. Table 6 shows the functional gene network analysis results, where column “ ♯ Edges” indicates the numbers of edges in the estimated gene networks of specific cancer cell lines.

**TABLE 6:** Multivariate gene network enrichment analysis results for immune disease pathways across cancer cell lines.

Cancer type	♯ edges	mES	mNES	p -value	FDR q -value
Colorectal	41,318	−0.289	−1.831	0.010	0.020
Pancreatic	31,120	−0.383	−2.327	0.000	0.000
Gastric	23,579	−0.249	−1.577	0.040	0.048
Breast	35,050	−0.113	−0.662	0.457	0.457
SCL	29,575	−0.263	−1.607	0.035	0.048
NSCL	56,621	−0.317	−1.977	0.000	0.000

As shown in Table 6, NSCL and colorectal cancer cell networks exhibited relatively dense molecular interplay, characterized by numerous edges, whereas gastric cancer showed a notably sparser network structure. These results demonstrate significant enrichment of the immune disease pathways in pancreatic cancer and NSCLC at the 0.01 significance level (α=0.01) , with both cancer types exhibiting strong negative mES values. Furthermore, immune disease-related genes tended to be systematically dysregulated in these cancers, reflecting substantial immune-related network rewiring compared to normal cell lines. Although colorectal, gastric, and SCL cancers also showed moderate associations, these signals did not remain significant under the more stringent threshold of significance level, α=0.01 , indicating weaker or less consistent immune pathway perturbations across these cancer types. Interestingly, breast cancer showed no significant enrichment, implying that immune disease-associated functional pathways do not represent a dominantly dysregulated network signature.

**FIGURE 4:**
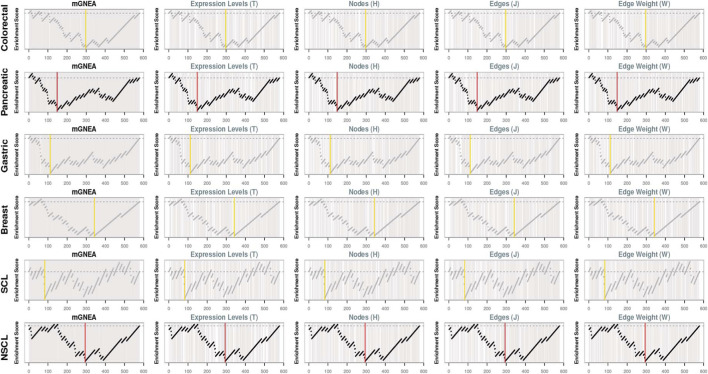
Network enrichment of immune disease pathways associated with cancer progression.

Figure 4 presents the enrichment score patterns. The columns “mFGNA,” “Expression Levels (T),” “Node (H),” “Edge (J),” and “Edge Weight (W)” correspond to the proposed multivariate enrichment score and the single-feature enrichment scores derived from expression, node centrality, edge structure, and edge-weight information, respectively. Genes involved in immune disease pathways were overrepresented at the lower end of the ranked gene list derived from pancreatic cancer and NSCLC versus normal gene networks (Figure 4). This pattern indicated that immune disease pathway genes contributed disproportionately to the observed network-level dysregulation.

**FIGURE 5:**
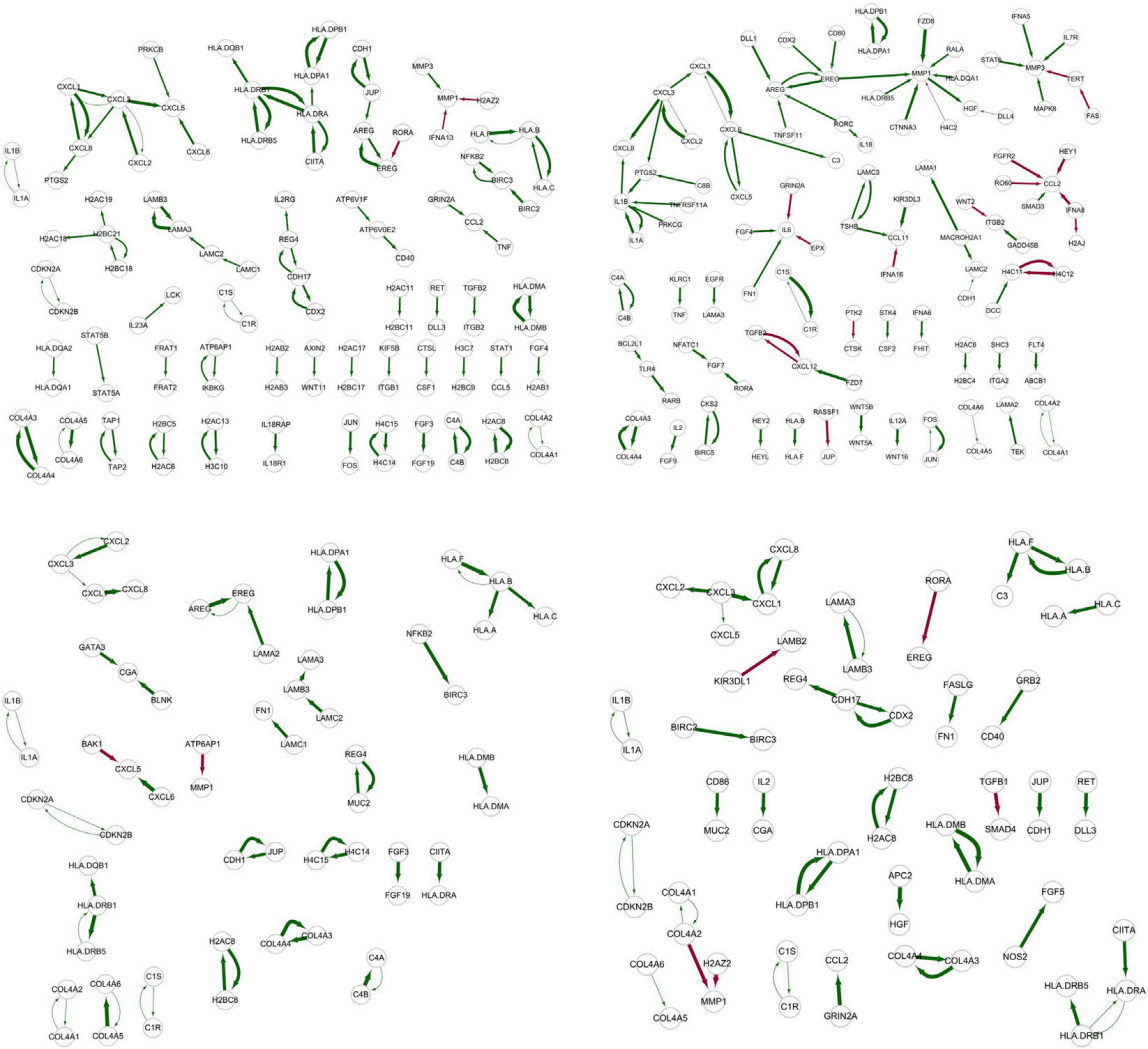
Gene networks in the identified significantly enriched cancer types (i.e., non-small-cell lung cancer (NSCLC) and pancreatic cancer) versus normal cell lines, highlighting enrichment in immune disease–associated pathways. The upper panels show the combined cancer network and the corresponding normal-cell network, whereas the lower panels display NSCLC-specific and pancreatic cancer–specific networks inferred separately. Edge colors indicate positive (green) or negative (red) regulatory effects from ⊕ to ⊗ (i.e., ⊕→⊗ ), while edge thickness represents the strength of the regulatory effect (i.e., edge weights).

To uncover immune-disease-related molecular interactions associated with cancer, we constructed gene networks of cancers significantly associated with immune disease pathways (i.e., NSCL and pancreatic cancers) and extracted the edges common to both networks. We then defined a shared immune disease gene network for both based on assignment of edge weights as the average of the corresponding edge weights in the two networks. The upper panels of Figure 5 show the combined immune disease–associated gene network constructed from NSCLC and pancreatic cancer cell lines, together with the corresponding network of normal cell lines, thereby highlighting molecular interaction patterns shared across the two significantly enriched cancer types. For clearer visualization of the complex network structures, the top 100 gene–gene interactions corresponding to the largest absolute values of their edge weights were focused on. Notably, immune disease gene networks exhibited different molecular interplay patterns in cancer cell lines compared to normal cell lines (Figure 5). The subnetwork of members of the C-X-C motif chemokine ligand (CXCL) family (i.e., interactions among CXCL1, CXCL2, CXCL3, CXCL5, and CXCL6) was found to be a common molecular interaction pattern shared by both cancer and normal cell lines. Interactions between HLA class II, that is, HLA-DR, HLA-DP, and HLA-DQ families, represented cancer-specific molecular interactions. Specifically, the HLA subnetwork constituted a major interaction module in cancer cell lines, whereas these interactions were largely absent in normal cell lines, except for those involving the HLA-DP family. Furthermore, HLA-DRA emerged as a key regulator in cancer networks, but its activity was not observed in normal networks.

In contrast, the hubness of MMP1, MMP3, CCL2, and IL1B represented normal cell-specific molecular characteristics. These genes are well-known mediators of extracellular matrix remodeling and inflammation, suggesting that normal cell networks may preserve coordinated tissue maintenance and immune-response pathways (Guan et al., 2025). Importantly, this hubness appeared to arise not from their regulatory influence over other genes but rather from being highly regulated by upstream factors. Overall, these results indicate that these genes function primarily as downstream responsive targets within normal cellular programs, in contrast to the cancer-specific emergence of active regulatory hubs.

**FIGURE 6:**
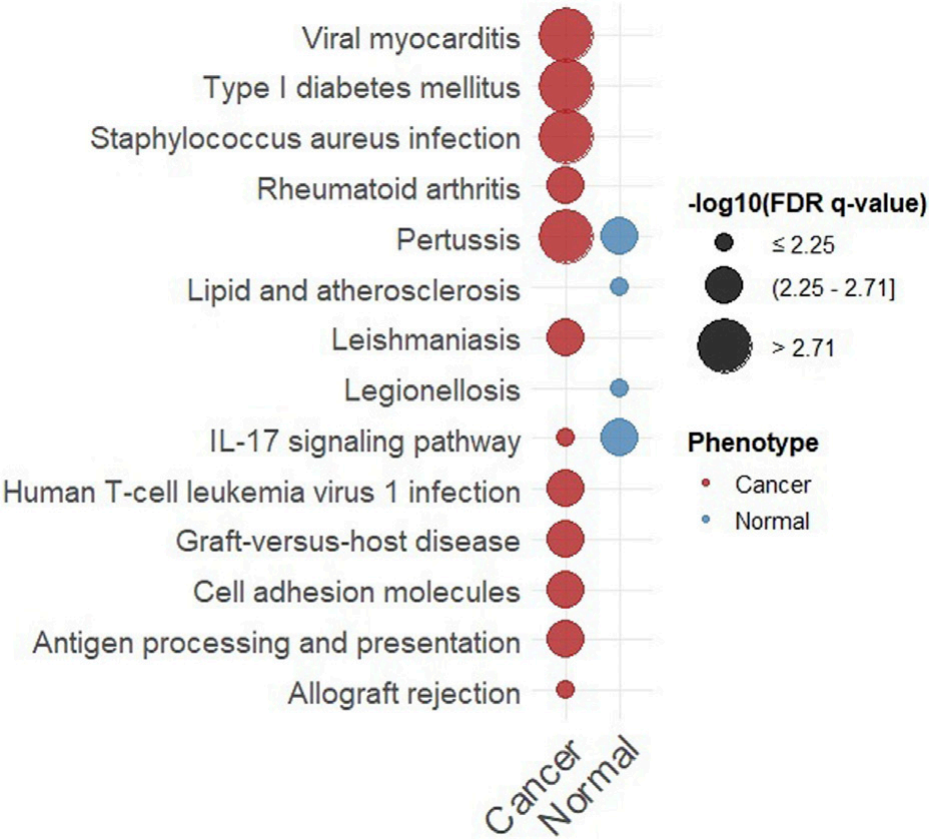
Kyoto Encyclopedia of Genes and Genomes (KEGG) pathway analysis results for immune disease-associated gene networks in non-small-cell lung cancer and pancreatic cancer (Cancer) and normal cell lines (Normal). Rows correspond to enriched pathways, and columns indicate the phenotypes. Bubble size represents enrichment significance, measured as -log10(FDR q-value), whereas bubble color distinguishes the phenotypes (Cancer and Normal).

**FIGURE 7:**
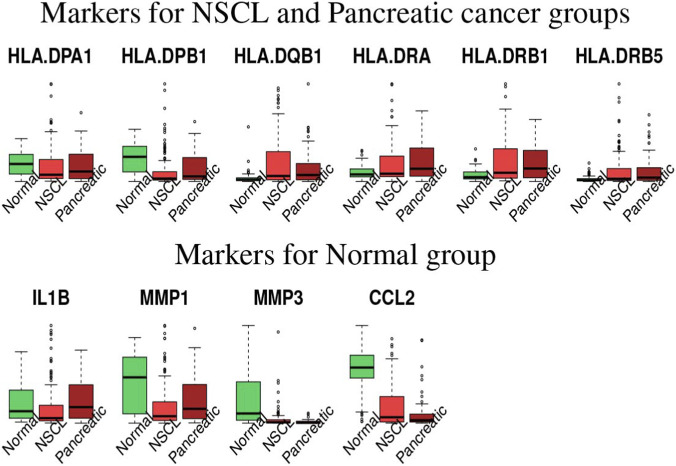
Expression of the identified markers for Non-small-cell lung/Pancreastic cancers and normal groups. Box plots illustrating the median and distribution of expression of the makers in normal (green), NSCLC (orange) and Pancreatic cancer (red) groups.

KEGG analysis was performed to investigate the biological functions of the enriched immune disease-associated gene networks in cancer and normal cell lines using DAVID (Dennis et al., 2003). The genes comprising the networks shown in Figure 5 were subjected to pathway analysis, and 332 immune disease genes were used as background genes. Figure 6 shows the significantly enriched KEGG pathways for the networks (FDR.q-value ≤0.01 ), where the bubble color and size denote enrichment significance, that is, –log(FDR.q-value). Rows correspond to enriched pathways and columns indicate phenotypes, that is, cancer (i.e., NSCL and pancreatic cancers) and normal cell lines. Notably, immune disease-associated molecular interactions in NSCL and pancreatic cancers were enriched across various KEGG pathways compared to those observed in normal cell lines (Figure 6). The Pertussis and IL-17 signaling pathways, which were the most enriched pathways in the normal group, were common to both cancer and normal cell lines. Additionally, the results showed that several pathways, including Type I diabetes mellitus, *Staphylococcus aureus* infection, Viral myocarditis, and Graft-versus-host disease, were significantly and preferentially enriched in the immune disease gene networks of NSCL and pancreatic cancer, underscoring cancer-specific immune dysregulation. Collectively, our findings suggest that dysregulation of normal-specific immune genes and cancer-associated immune markers may reflect cancer-associated immune regulatory alterations observed in cancer cell lines and provide hypotheses regarding mechanisms potentially involved in cancer progression. Figure 7 illustrates the expression of the identified immune disease markers in normal, NSCLC, and pancreatic cancer cell lines. Immune disease–associated cancer markers, including HLA-DQB1, HLA-DRA, HLA-DRB1, and HLA-DRB5, were upregulated in NSCLC and pancreatic cancer cell lines, suggesting altered antigen presentation–related programs in these cancer cell models. Moreover, normal-cell-specific markers (IL1B, MMP1, MMP3, and CCL2) displayed a more distinct expression pattern, with consistently higher expression in normal cell lines than in cancer cell lines. This suggested that inflammatory and extracellular matrix–related signaling may be more prominent under normal conditions, whereas cancer cells preferentially exhibit immune disease–associated alterations linked to antigen processing and presentation.

To further investigate the characteristics of each cancer type, we additionally inferred and visualized NSCL-specific and pancreatic cancer–specific immune disease–associated gene networks separately in the lower panels of Figure 5. The cancer-specific networks revealed both shared and distinct molecular interaction patterns between NSCL and pancreatic cancers. Consistent with the combined cancer network shown in the upper panels, interactions among HLA class II genes, including members of the HLA-DR, HLA-DP, and HLA-DQ families, were observed in both NSCL- and pancreatic cancers -specific networks, suggesting that dysregulation of antigen presentation machinery represents a common immune-related characteristic across the two cancer types. However, several molecular interactions appeared to be cancer-type specific. Although both NSCLC- and pancreatic cancer-specific networks retained core immune-related interaction modules, the NSCLC-specific network uniquely contained interactions involving H2AC6–H2BC3 and MUC2–REG4, whereas the pancreatic cancer-specific network exhibited distinct interactions involving TGFB1–SMAD4 and NOS2–FGF5. These cancer-specific interactions suggest that different regulatory mechanisms may contribute to immune pathway dysregulation in the two cancer types. Therefore, while the combined cancer network summarizes immune-related interactions shared across NSCLC and pancreatic cancer, the cancer-specific networks reveal distinct molecular interaction patterns that would otherwise be obscured in the merged network.

## 4 Discussion

In this study, we proposed mFGNA, a novel multivariate framework for functional GNEA, which integrates multiple topological features, including node centrality, edge structure, and edge weights, along with traditional expression to address the limitations of conventional GNEA. In contrast to the existing GNEA methods, which often suffer from information loss by aggregating features into a single composite score, mFGNA preserves such multi-dimensional characteristics, thereby enhancing biological interpretability. Furthermore, mFGNA employs gene-level permutations to construct a null distribution consistent with the enrichment hypothesis, thus addressing the inferential inconsistency of phenotype-based permutations. This contributes to a more robust significance evaluation in functional GNEA.

The Monte Carlo simulations showed that mFGNA consistently outperformed conventional approaches, such as GbNEA, NEAT, ORA, and GSEA-based approaches, regarding discriminative power and robustness across various network sizes and cancer types. Application of mGNEA to immune disease gene networks further revealed pronounced immune pathway dysregulation across multiple cancer types. Cancer-specific interactions were dominated by HLA class II genes (HLA-DR, HLA-DP, and HLA-DQ), forming dense subnetworks; they were largely absent in normal networks. Biologically, the HLA-DR, HLA-DP, and HLA-DQ families are the core components of the major histocompatibility complex class II antigen presentation machinery, which plays a central role in activating CD4^+^ T cell–mediated immune responses (Seliger et al., 2017). Thus, cancer-specific formation of a densely connected HLA interaction module suggests substantial immune regulatory rewiring in tumor cells. In contrast, normal cell-specific hub genes such as MMP1, MMP3, and CCL2 exhibited distinct regulatory patterns and were primarily influenced by upstream components. The markers identified by the proposed strategy have previously been implicated in cancer progression, immune regulation, and tumor microenvironment remodeling.

- HLA class II: HLA-DR, HLA-DP, and HLA-DQ
  HLA class II expression is regulated by CIITA and IFN- γ signaling but is often disrupted in tumor cells due to structural, epigenetic, or post-translational alterations (Barbeau et al., 2025). Barbeau et al. (2025) associated low MAP1LC3C expression in NSCLC with reduced CIITA and HLA class II expression, along with decreased immune cell infiltration. Additionally, Datar et al. (2021) showed that immunotherapy-naive NSCLCs frequently exhibited defects in HLA class II subunit expression, which are linked to distinct tumor microenvironment profiles and patient survival outcomes.

- MMP1 and MMP3
Lu et al. (2025) suggested that tumor-derived transforming growth factor- β modulates PSC matrix metalloproteinase (MMP) profiles, suppressing pancreatic cancer cell migration, and highlighting MMPs as potential diagnostic markers. Liang et al. (2021) reported that tumor cell–associated MMP3 promotes tumor growth and invasion, positioning MMP3, particularly in combination with oncolytic virus therapy, as a promising anticancer strategy. Moreover, Wang et al. (2020) revealed that MMP-1 facilitated colorectal cancer progression through epithelial–mesenchymal transition (EMT) and the p-Akt signaling pathway.

- CCL2
  Various tumor types produce C-C Motif Chemokine Ligand (CCL)2, which plays a key role in cancer progression by promoting tumor cell migration and invasion (Li et al., 2018). Hajibabaie et al. (2026) highlighted the contribution of CCL2 to colorectal cancer via tumor-associated macrophage and immune cell recruitment, and activation of the CCL2–CCR2 axis promoted EMT and enhanced tumor invasion. Consistently, Xu et al. (2021) reported that the CCL2–CCR2 signaling pathway regulates diverse tumor cell activities across tumorigenesis. Moreover, Li et al. (2018) suggested that CCL2 and CCL4 expression may serve as prognostic biomarkers and therapeutic targets in NSCLC.

- IL1B
  High interleukin (IL)-1 beta (IL1B) and IL-1 receptor, type I expression has been identified as a therapy response marker in patients with NSCLC receiving chemo-immunotherapy (Perrichet et al., 2025). IL1B signaling contributes to the inflammatory component of cancer (Garon et al., 2020); Khawkhiaw et al. (2024) showed the central role of IL1B in tumor-promoting activities across many malignancies, including gastrointestinal cancers. Notably, IL1B may exert anti-tumor effects by activating and recruiting immune cells into the tumor microenvironment, depending on the immune context.

We suggest through our results and prior literature that the activation of normal-specific immune genes (i.e., MMP1, MMP3, and CCL2), together with the aberrant upregulation of cancer-associated immune markers (i.e., HLA class II genes), may provide crucial mechanistic insights into the immune-driven processes underlying cancer progression. These findings suggest that modulation of HLA class II–related immune pathways may represent a potential avenue for therapeutic intervention and warrant further investigation. Particularly, these findings provide hypotheses regarding molecular pathways that may be relevant to immune regulation in cancer and warrant further biological validation. Overall, These results suggest that normal networks emphasized coordinated tissue maintenance and chemokine signaling, whereas cancer networks shifted toward antigen-presentation–driven immune signatures. These findings highlight immune remodeling in tumors and indicate that aberrant antigen-processing networks represent key features of the tumor immune microenvironment, with potential implications for immune-modulatory strategies targeting cancer progression.

In conclusion, mFGNA provides a powerful and comprehensive framework for the identification of functional pathways driven by complex molecular interactions. In this study, the identified immune-related network markers offer valuable implications for the discovery of diagnostic biomarkers and design of therapeutic strategies targeting tumor–immune microenvironment. By integrating diverse network-level features, while preserving their distinct contributions, the proposed approach facilitates a deeper understanding of pathway dysregulation in complex diseases, and highlights potential molecular targets for future therapeutic interventions.

Several limitations of this study should be acknowledged. Although the proposed framework was evaluated using both DepMap and TCGA datasets, these analyses should be regarded as real-data benchmarking experiments rather than fully controlled simulations with known network ground truth. Specifically, the true-positive and true-negative scenarios were constructed using biologically curated pathway annotations and experimentally measured transcriptomic data, and therefore the underlying network structures were not known *a priori*. While this design provides a biologically realistic evaluation framework, it does not permit direct assessment of network reconstruction accuracy. Furthermore, real-world biological datasets often exhibit substantial heterogeneity, technical variability, batch effects, and measurement noise that may not be fully captured by the current benchmarking settings. Consequently, the performance of mFGNA in other biological contexts or datasets may differ from that reported in this study. Future work should therefore evaluate the proposed framework using additional independent cohorts, experimentally validated gene networks, and diverse multi-omics datasets to further assess its robustness and generalizability.

## Data Availability

The implementation code and accompanying toy data for mFGNA have been deposited in Figshare and can be accessed via https://doi.org/10.6084/m9.figshare.31369045.

## References

[B1] Barbeau L. M. O. Beelen N. A. Savelkouls K. G. Keulers T. G. H. Wieten L. Rouschop K. M. A. (2025). MAP1LC3C repression reduces CIITA- and HLA class II expression in non-small cell lung cancer. PLoS One 20 (2), e0316716. 10.1371/journal.pone.0316716 39928678 PMC11809862

[B2] Benjamini Y. Hochberg Y. (1995). Controlling the false discovery rate: a practical and powerful approach to multiple testing. J. R. Stat. Soc. Ser. B 57, 289–300. 10.1111/j.2517-6161.1995.tb02031.x

[B3] Chang L. Y. Lee M. Z. Wu Y. Lee W. K. Ma C. L. Chang J. M. (2024). Gene set correlation enrichment analysis for interpreting and annotating gene expression profiles. Nucleic Acids Res. 52, e17. 10.1093/nar/gkad1187 38096046 PMC10853793

[B4] Cheng C. W. Beech D. J. Wheatcroft S. B. (2020). Advantages of CEMiTool for gene co-expression analysis of RNA-seq data. Comput. Biol. Med. 125, 103975. 10.1016/j.compbiomed.2020.103975 32911277

[B5] Datar I. J. Hauc S. C. Desai S. Gianino N. Henick B. Liu Y. (2021). Spatial analysis and clinical significance of HLA Class-I and Class-II subunit expression in non-small cell lung cancer. Clin. Cancer Res. 27 (10), 2837–2847. 10.1158/1078-0432.ccr-20-3655 33602682 PMC8734284

[B6] DennisG. ShermanB. T. HosackD. A. YangJ. GaoW. LaneH. C. (2003). DAVID: database for annotation, visualization, and integrated discovery. Genome Biol. 4, P3. 12734009

[B7] Draghici S. Khatri P. Martins R. P. Ostermeier G. C. Krawetz S. A. (2003). Global functional profiling of gene expression. Genomics 81, 98–104. 10.1016/s0888-7543(02)00021-6 12620386

[B8] Garon E. B. Chih-Hsin Yang J. Dubinett S. M. (2020). The role of interleukin 1*β* in the pathogenesis of lung cancer. JTO Clin. Res. Rep. 1 (1), 100001. 10.1016/j.jtocrr.2020.100001 34589908 PMC8474414

[B9] Guan F. Wang R. Yi Z. Luo P. Liu W. Xie Y. (2025). Tissue macrophages: origin, heterogeneity, biological functions, diseases and therapeutic targets. Signal Transduct. Target Ther. 10 (1), 93. 10.1038/s41392-025-02124-y 40055311 PMC11889221

[B10] Hajibabaie F. Abedpoor N. Maleki B. Boshtam M. Shenavar N. Zarrabi A. (2026). Targeting the CCL2/CCR2 axis in colorectal cancer: immunopharmacological perspectives and therapeutic strategies. Crit. Rev. Oncol. Hematol. 218, 105072. 10.1016/j.critrevonc.2025.105072 41360349

[B11] Jung D. (2024). gsean: gene set enrichment analysis using networks. R. Package Version 1.26.0. 1–8.

[B12] Khawkhiaw K. Panaampon J. Imemkamon T. Saengboonmee C. (2024). Interleukin-1*β*: friend or foe for gastrointestinal cancers. World J. Gastrointest. Oncol. 16 (5), 1676–1682. 10.4251/wjgo.v16.i5.1676 38764841 PMC11099428

[B13] Li Y. Luo P. Wu C. (2014). A new network node similarity measure method and its applications. arXiv 1403, 4303.

[B14] Li L. Liu Y. D. Zhan Y. T. Zhu Y. H. Li Y. Xie D. (2018). High levels of CCL2 or CCL4 in the tumor microenvironment predict unfavorable survival in lung adenocarcinoma. Thorac. Cancer 9 (7), 775–784. 10.1111/1759-7714.12643 29722145 PMC6026602

[B15] Liang M. Wang J. Wu C. Wu M. Hu J. Dai J. (2021). Targeting matrix metalloproteinase MMP3 greatly enhances oncolytic virus mediated tumor therapy. Transl. Oncol. 14 (12), 101221. 10.1016/j.tranon.2021.101221 34530193 PMC8450250

[B16] Liu Y. Liang J. Zhang Y. Guo Q. (2024). Drug resistance and tumor immune microenvironment: an overview of current understandings. Int. J. Oncol. 65 (4), 96. 10.3892/ijo.2024.5684 39219258 PMC11387120

[B17] Lu M. Jiang D. Zhang J. Liu L. Shen Y. Li T. (2025). Diagnostic valuation of MMP family members as biomarkers for pancreatic cancer: a systematic review and meta-analysis. Biomark. Med. 19 (18), 909–919. 10.1080/17520363.2025.2571394 41105435 PMC12542613

[B18] Park H. Imoto S. Miyano S. (2025). Gene behaviors-based network enrichment analysis and its application in revealing immune disease pathways enriched with COVID-19 severity-specific gene networks. Bioinformatics 41 (7), btaf378. 10.1093/bioinformatics/btaf378 40580453 PMC12270264

[B19] Perrichet A. Lecuelle J. Limagne E. Thiefin M. Bellio H. Jacob P. (2025). Cancer cell-derived IL-1*β* reverses chemo-immunotherapy resistance in non-small cell lung cancer. Nat. Commun. 16 (1), 10244. 10.1038/s41467-025-64839-4 41271647 PMC12639146

[B20] Seliger B. Kloor M. Ferrone S. (2017). HLA class II antigen-processing pathway in tumors: molecular defects and clinical relevance. Oncoimmunology 6 (2), e1171447. 10.1080/2162402X.2016.1171447 28344859 PMC5353941

[B21] Signorelli M. Vinciotti V. Wit E. C. (2016). NEAT: an efficient network enrichment analysis test. BMC Bioinforma. 17 (1), 352. 10.1186/s12859-016-1203-6 27597310 PMC5011912

[B22] Subramanian A. Tamayo P. Mootha V. K. Mukherjee S. Ebert B. L. Gillette M. A. (2005). Gene set enrichment analysis: a knowledge-based approach for interpreting genome-wide expression profiles. Proc. Natl. Acad. Sci. U. S. A. 102, 15545–15550. 10.1073/pnas.0506580102 16199517 PMC1239896

[B23] Tian L. Greenberg S. A. Kong S. W. Altschuler J. Kohane I. S. Park P. J. (2005). Discovering statistically significant pathways in expression profiling studies. Proc. Natl. Acad. Sci. U. S. A. 102 (38), 13544–13559. 10.1073/pnas.0506577102 16174746 PMC1200092

[B24] Tibshirani R. (1996). Regression shrinkage and selection via the lasso. J. R. Stat. Soc. Ser. B 58, 267–288. 10.1111/j.2517-6161.1996.tb02080.x

[B25] Tiong K. L. Yeang C. H. (2019). MGSEA - a multivariate gene set enrichment analysis. BMC Bioinforma. 20 (1), 145. 10.1186/s12859-019-2716-6 30885118 PMC6421703

[B26] Tufail M. Jiang C. H. Li N. (2025). Immune evasion in cancer: mechanisms and cutting-edge therapeutic approaches. Signal Transduct. Target Ther. 10 (1), 227. 10.1038/s41392-025-02280-1 40739089 PMC12311175

[B27] Vengatharajuloo V. Goh H. H. Hassan M. Govender N. Sulaiman S. Afiqah-Aleng N. (2023). Gene Co-Expression network analysis reveals key regulatory genes in Metisa plana hormone pathways. Insects 14 (6), 503. 10.3390/insects14060503 37367319 PMC10299145

[B28] Wang K. Zheng J. Yu J. Wu Y. Guo J. Xu Z. (2020). Knockdown of MMP-1 inhibits the progression of colorectal cancer by suppressing the PI3K/Akt/c-myc signaling pathway and EMT. Oncol. Rep. 43 (4), 1103–1112. 10.3892/or.2020.7490 32323782 PMC7057971

[B29] Wang X. Bajpai A. K. Gu Q. Ashbrook D. G. Starlard-Davenport A. Lu L. (2023). Weighted gene co-expression network analysis identifies key hub genes and pathways in acute myeloid leukemia. Front. Genet. 14, 1009462. 10.3389/fgene.2023.1009462 36923792 PMC10008864

[B30] Xu M. Wang Y. Xia R. Wei X. (2021). Role of the CCL2-CCR2 signalling axis in cancer: mechanisms and therapeutic targeting. Cell Prolif. 54 (10), e13115. 10.1111/cpr.13115 34464477 PMC8488570

[B31] Zou H. Hastie T. (2005). Regularization and variable selection via the elastic net. J. R. Stat. Soc. Ser. B 67, 301–320. 10.1111/j.1467-9868.2005.00503.x

[B32] Zou H. Hastie T. Tibshirani R. (2007). On the “degrees of freedom” of the lasso. Ann. Stat. 35 (5), 2173–2192. 10.1214/009053607000000127

